# Toward vanishing droplet friction on repellent surfaces

**DOI:** 10.1073/pnas.2315214121

**Published:** 2024-04-15

**Authors:** Matilda Backholm, Tytti Kärki, Heikki A. Nurmi, Maja Vuckovac, Valtteri Turkki, Sakari Lepikko, Ville Jokinen, David Quéré, Jaakko V. I. Timonen, Robin H. A. Ras

**Affiliations:** ^a^Department of Applied Physics, Aalto University, Espoo 02150, Finland; ^b^Centre of Excellence in Life-Inspired Hybrid Materials, Aalto University, Espoo 02150, Finland; ^c^Department of Chemistry and Materials Science, Aalto University, Espoo 02150, Finland; ^d^Physique et Mécanique des Milieux Hétérogènes, UMR 7636 du CNRS, Paris Sciences Lettres Research University, Ecole Supérieure de Physique et Chimie Industrielles, Paris 75005, France

**Keywords:** drop friction, superhydrophobic, wetting

## Abstract

Superhydrophobic surfaces are often seen as frictionless materials, on which water is highly mobile. Understanding the nature of friction for such water-repellent systems is central to further minimize resistance to motion and energy loss in applications. For slowly moving drops, contact-line friction has been generally considered dominant on slippery superhydrophobic surfaces. Here, we show that this general rule applies only at very low speed. Using a micropipette force sensor in an oscillating mode, we measure the friction of water drops approaching or even equaling zero contact-line friction. We evidence that dissipation then mainly stems from the viscous shearing of the air film (plastron) trapped under the liquid. Because this force is velocity dependent, it can become a serious drag on surfaces that look highly slippery from quasi-static tests. The plastron thickness is found to be the key parameter that enables the control of this special friction, which is useful information for designing the next generation of ultraslippery water-repellent coatings.

## Significance

A small raindrop will easily slide off the surface of a lotus leaf or the feathers of a bird. These natural systems are beautiful examples of superhydrophobic surfaces. Today, there is a strong focus on mimicking this natural surface design to manufacture artificial liquid-repellent substrates for a wide range of applications, such as self-cleaning and nonwetting materials. The physics describing how water drops move on these surfaces has been studied extensively to find even better surface designs. Here, we developed a technique to measure the tiny friction of drops moving on extremely slippery superhydrophobic surfaces. We found an important plastron friction force that needs to be considered when designing the next generation of ultraslippery water-repellent coatings.

**Fig. 1.**
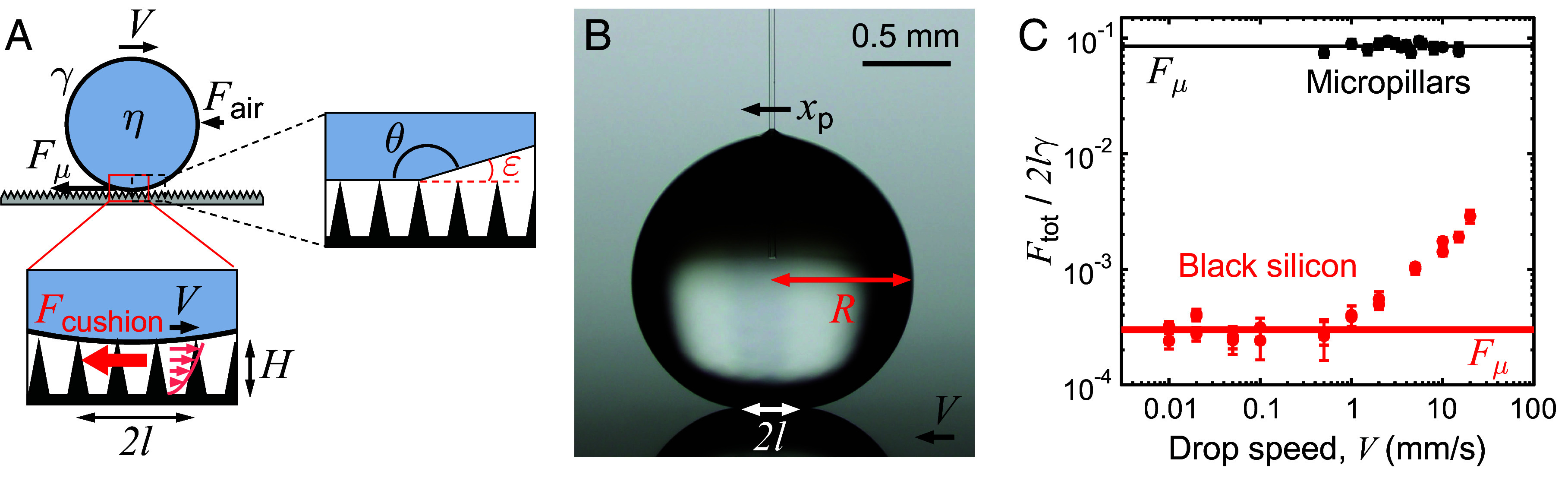
Friction mechanisms. (*A*) A drop (radius *R*, contact radius *l*, surface tension γ, viscosity η, contact angle θ, and wedge angle ε=π-θ) moving at speed *V* on a superhydrophobic surface is believed to be mainly opposed by contact-line friction $F_{\mu }$, viscous force $F_{\eta }$ caused by the motion of the drop, and air resistance $F_{air}$. However, the moving drop also shears the air trapped within the microstructured coating (thickness H), which generates a viscous force $F_{cushion}$ in this plastron (*Inset* at the *Bottom*). Schematic drawings not to scale. (*B*) Photo of a water drop on black silicon in motion and probed with a micropipette force sensor (MFS). The substrate is moved to the left and the total friction force is deduced from the deflection $x_{p}$ of the micropipette: *F*_tot_ $=k_{p}x_{p}$, denoting *k*_p_ as the micropipette stiffness. (*C*) Total friction force *F*_tot_ measured by MFS and normalized by 2γ*l*, as a function of the drop speed *V*. Black and red data are obtained on superhydrophobic micropillars and black silicon, respectively. In the latter case, friction unexpectedly starts to rise at drop speeds V<2 mm/s. Error bars are error propagations of the SD from the MFS experiment.

Drops moving on liquid-repellent surfaces is a common phenomenon in everyday life, with water pearls running down plant leaves (1) or across hot frying pans (2). Understanding the many different frictions (Fig. 1*A*) opposing motion in these systems (3–11) is important for improving the quality and usability of a wide range of liquid-repellent coatings (12, 13). At low drop speeds (V≲1 mm/s), at which velocity-dependent resistances are all expected to vanish, water drops (of surface tension γ, density ρ, and viscosity η) are still impeded by the contact-line friction force $F_{\mu }\approx 2l\gamma (\cos\theta_{rec}-\cos\theta_{adv}$) (5, 7, 9, 14–17), where *l* is the contact area radius, and $\theta_{rec}$ and $\theta_{adv}$ are the receding and advancing contact angles (*SI Appendix*, Fig. S1). For a drop with radius R≈1 mm, both the capillary number Ca=ηV/γ and the Weber number $We=\rho V^{2}R/\gamma$ are smaller than $10^{-5}$, implying that drops keep a quasi-static shape with contact size *l* independent of *V*, despite the motion.

The contact-line friction itself can become small on highly superhydrophobic surfaces (9, 18) with high contact angles θ and thus small l and low hysteresis $\theta_{\text{adv}}-\;\theta_{\mathrm{rec}}$. The dimensionless total friction $F_{tot}/2l\gamma$ opposing the motion can be directly measured with a MFS (9, 18–20) (Fig. 1*B*). In this technique, drops are scanned over a moving substrate using a calibrated glass cantilever whose deflection provides the friction force (*Materials and Methods* and *SI Appendix*, Fig. S2). As seen in Fig. 1*C* (black data), friction at low speed on conventional hydrophobic micropillars (*SI Appendix*, Fig. S3) is indeed found to be independent of *V*, and at a dimensionless friction value of 0.1 expected for $\theta_{\text{adv}}\approx 165^{\circ }\;\text{and}\;\theta_{\mathrm{rec}}\approx 150^{\circ }$. Previous experiments on highly slippery “black silicon” (bSi) surfaces show a similar behavior for V<2 mm/s (9), yet with a friction divided by more than 100 (red data in Fig. 1*C*), owing to the ultraslippery nature of these substrates. The absolute value of the friction $F_{tot}$ is typically 10 nN, a tiny quantity compared to the drop weight, of order 10 µN.

However, as seen in Fig. 1*C*, data at higher speed (V<2 mm/s) become velocity dependent, with a tenfold increase in force when the speed increases from 2 mm/s to 2 cm/s. A seemingly similar increase was reported by Gao et al. (7) for hexadecane and ionic liquid on fluorinated (non-superhydrophobic) silicon and interpreted as a consequence of a velocity-dependent contact angle hysteresis Δcosθ(V) at “high” drop speed (21). However, significant variations of the hysteresis are only expected at large enough capillary number (Ca > 10^−3^), far above the range of our experiments. We confirmed this point by measuring the hysteresis of water on black silicon, ca $0.4^{\circ },$ a quantity found to be independent of the drop speed (*SI Appendix*, Fig. S1 *B* and *C*). In addition, this low value provides a contact-line friction (normalized by 2γ*l*) smaller than 10^−3^ at high angle, in agreement with the data at low speed in Fig. 1*C*. Hence, we conclude that another velocity-dependent force is revealed by the ultraslippery nature of the substrate, in this intermediate regime of drop speed. We try here to discuss the origin of this force, an important step to understand the high mobility of drops on repellent materials (1, 8, 11, 22, 23).

In this paper, we probe this system in depth, which we achieve by developing an oscillating MFS technique. This provides a direct measurement of the friction force and damping coefficient of drops on repellent surfaces in two complementary cases, either with a strictly zero contact-line friction or on slippery black silicon. In the first case, we consider a carbonated water drop levitating $\left(F_{\mu }=0\right)$ on a transparent superhydrophobic material. In the second case, we study water drops moving on black silicon with various texture heights, yet with constant contact angles and hysteresis, which eventually confirms the existence of a previously disregarded friction for water drops moving on repellent materials. We finally introduce a sample design usable in the future to minimize this source of resistance.

## Results

### Friction Forces.

In addition to contact-line friction, drop motion can be influenced by electrostatic forces (11), aerodynamic resistance (8), and viscous resistance inside the drop (3, 4, 8). The two first effects should be negligible in our case. First, we did not detect any sign of electrostatic forces in our MFS measurements, with no difference in the forces as a function of scanning distance or after changing the drop for a new one. Second, it is known that aerodynamic friction is only relevant when the drop speed is a few tens of cm/s, far above the range explored in this study (8). Denoting η_a_ and ρ_a_ as the air viscosity and density, viscous and inertial frictions in air respectively scale as η_a_*VR* and ρ_a_*V*^2^*R*^2^, both on the order of 0.1 nN for *V* = 1 cm/s, far below the frictions reported in Fig. 1*C*. A more subtle air friction takes place in the wedge of air present around the drop. Following Scriven’s analysis (24, 25) and denoting ε=π-θ as the wedge angle (Fig. 1*A*), we expect this friction to scale as η_a_*Vl*/ε ln(*R*/*H*), where the logarithmic factor accounts for the singular character of the friction in the wedge, but also from the fact that air can slip on the air trapped in the texture with height *H*. This force is typically around 1 nN, ten times larger than the viscous friction in air, but small compared to our data. In addition, it can be tested in our experiment, in particular as a function of the contact area radius *l*, to which it is expected to be proportional. As emphasized by Mahadevan and Pomeau (8), a nonwetting contact results from a balance between weight and surface tension, which yields *l* ~ *R*^2^/l_c_, where l_c_ = (γ/ρ*g*)^1/2^ is the capillary length (2.7 mm for water). All drops in this study will be smaller than the capillary length, that is, rounded by surface tension rather than flattened by gravity—but they will keep a gravity-induced contact *l* < *R*, a crucial point for understanding their friction.

The viscous friction in the drop is also natural to consider (3, 4, 8). At high liquid viscosity η, we expect the viscous friction to scale at most as (η*V*/*R*)*l*^2^, that is, η*Vl*^3/2^/l_c_^1/2^ after using the Mahadevan–Pomeau law of contact (3, 4, 8). For water (η=1 mPa.s), this force is expected to be on the order of 1 nN, again significantly smaller than the force of about 10 nN measured in the velocity-dependent regime in Fig. 1*C*. This law, however, can be questioned for a liquid with low viscosity such as water. Then, dissipation might rather occur in the viscous boundary layer localized in the contact zone. With a thickness δ scaling as (η*l*/ρ*V*)^1/2^, we find a force (η*V*/δ)*l*^2^ ~ η^1/2^ρ^1/2^*V *^ 3/2^*l *^3/2^, whose dependency remains in *l*^3/2^ and magnitude of order 1 nN. Hence, we conclude that the viscous friction in the drop is negligible compared to the observed one.

After eliminating these canonical sources of dynamic friction, we finally consider a force specific to repellent materials, arising from the viscous dissipation in the plastron of air trapped within the microtexture or present below drops when they levitate above their substrate. Here, the term “plastron” specifically refers to the air layer trapped within the microstructure of a superhydrophobic coating. In contrast to other air or gas films present in the levitation (2, 26–30) or bouncing (31) dynamics of drops, the plastron of superhydrophobic substrates remains stable for a long time also for stationary drops or submerged surfaces (32). The plastron is thus highly important to a broad range of wetting phenomena on superhydrophobic materials, including drop bouncing and splashing, but also steady drop sliding and rolling motion. Slippery surfaces have a minimized contact with drops, and they provide an ultralow contact angle hysteresis so that we consider that water can slip on the plastron. This generates velocity gradients on the order of *V*/*H* inside the plastron, assuming that the viscous boundary layer in air has invaded the whole texture, which it does at micrometric scales. The corresponding plastron friction thus scales as ${(\eta }_{a}V/H)l^{2}$, a quantity expected to be on the order of 10 nN for micrometric texture, consistent with our observations. This force exceeds line friction for *V* > γΔcosθ*H*/η_a_*l*, that is, around 1 m/s for Δcosθ = 10^−1^ and 1 cm/s for Δcosθ = 10^−3^, again in accordance with data in Fig. 1*C* where we only see a plateau for micropillars with high hysteresis (black data), contrasting with the deviations linear in velocity around 1 cm/s on black silicon of ultralow hysteresis (red data).

In the following sections, we present an experimental technique developed to directly probe the friction on such low-friction surfaces. This allows us to test the model by playing separately with the parameters *l*, *V,* and *H.* In addition, we can test the case of levitating drops for which we have an air cushion yet no contact line (*F_µ_* = 0), a situation where the cushion friction can be tested independently of the presence of a line friction.

### Oscillating MFS Measurements.

**Fig. 2.**
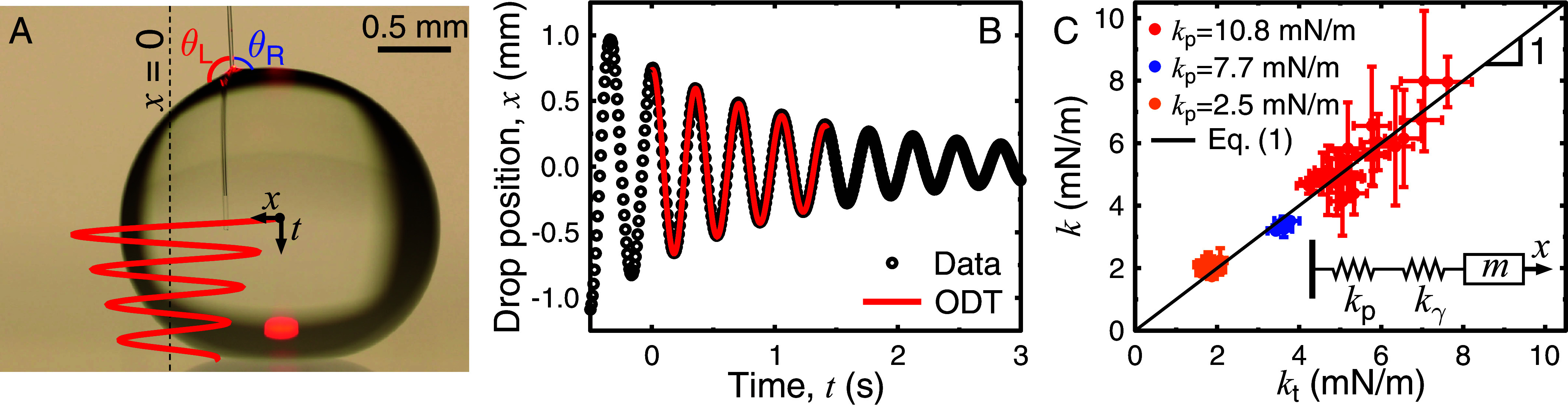
Oscillating MFS experiments. (*A*) A drop (here, carbonated water on Glaco-treated surface with R=1.12±0.03 mm and l=350±1 μm) is attached to a micropipette through capillary forces. The pipette is deflected by a distance $x_{p}$ from its equilibrium position *x* = 0 (dashed line) and released, leading to damped oscillations of the system (solid red line). The left- and right-hand contact angles between the drop and the micropipette are denoted as $\theta_{L}$ and $\theta_{R}$. (*B*) The center-of-mass drop position as a function of time. The oscillating droplet tribology (ODT) model with $F_{\mu }=0$ successfully fits the data for k=1.9±0.2 mN/m and β=7.4±0.7 µNs/m for the specific experiment shown in (*A*). (*C*) The spring constant *k* deduced from ODT is plotted as a function of the theoretically calculated spring constant *k*_t_ (Eq. **1**) for all oscillating MFS experiments performed on Glaco and black silicon, using three different micropipettes, each with its own spring constant *k*_p_. All data collapse onto a line with a slope of one, indicating excellent agreement between the data and model. The inset shows the series-connected springs of the oscillating MFS technique, where $k_{p}$ and $k_{\gamma }$ are the spring constants of the micropipette and the deformed drop interface, and *m* is the drop mass. The error bars are the 95% CI of the ODT fits for *k* as well as the error propagations of the components (and their SD) in Eq. **1** for *k*_t_.

In the oscillating MFS technique (Fig. 2*A*), a substrate-supported drop is attached through capillary forces at the end of a glass fiber (spring constant $k_{p}$, see *Materials and Methods* for experimental details). The drop-fiber device is deflected from the equilibrium position *x* = 0 and the subsequent oscillations are followed with a side-view camera, that gives the position *x* of the drop center of mass as a function of time *t* (Fig. 2*B*). The motion is opposed by an elastic force kx caused by the MFS cantilever, the line friction $F_{\mu }$ and a dissipative force we assume to be proportional to the velocity βV, as suggested by our first experiments. Denoting the drop mass as *m*, the solution of the equation of motion $m\left(d^{2}x/dt^{2}\right)=-kx-\beta V-F_{\mu }$ (33–36) is a damped harmonic oscillator function that successfully fits the data in Fig. 2*B*.

This oscillating droplet tribology approach has been robustly used for magnetic drops (33–35) and lubricated sliders (37) to probe wetting properties of superhydrophobic surfaces. Here we use the oscillating droplet tribometry (ODT) analysis methodology in the context of oscillating micropipettes. The ODT fit is performed for data with oscillation amplitudes that are small enough to be in the harmonic regime, but large enough to correspond to actual center-of-mass motion of the drop and not to the final “wiggling” motion of the drop interface. The outputs of the fit are *k*, $F_{\mu },$ and β. Since the contact-line friction force $F_{\mu }$ has been studied in detail in previous work (9), the damping coefficient β is the main focus of this study. As described in detail below, great care has been taken in experimentally and theoretically verifying the physical meaning of each of the three fitting parameters used by the ODT model. In addition, the ODT model was validated by measuring simulated data (see *SI Appendix*, Supplementary Text for more details).

The spring constant *k* of the drop-micropipette system can be described as a series of the spring-like micropipette, with known stiffness $k_{p}$, and the spring-like deformation of the drop interface at the micropipette-drop contact line (*Inset* in Fig. 2*C*). The spring constant $k_{\gamma }$ in the *x*-direction of the latter can be calculated by considering the left and right-hand contact angles $\theta_{L}$ and $\theta_{R}$ made by the drop with the micropipette (Fig. 2*A*): $k_{\gamma }=\gamma \left|\sin\theta_{L}-\sin\theta_{R}\right|$. This gives a total (theoretical) spring constant of

[1] $$k_{t}=\frac{1}{1/k_{p}+1/(\gamma \left|\text{sin}\theta_{L}-\text{sin}\theta_{R}\right|)}.$$

By analyzing the contact angles at the largest deflections of the drop-pipette system, at the beginning and end of the region used for the ODT fit, an average value of the surface tension–related spring constant was calculated and included in Eq. **1**. In Fig. 2*C*, the spring constant *k* from the ODT fits for all oscillating MFS data on Glaco and black silicon is plotted for three different micropipettes as a function of the spring constant *k*_t_ given by Eq. **1**. These two are in excellent agreement, as shown by the collapse of the data on a line with a slope one. This implies that neither *k*_t_ (described by Eq. **1**) nor *F*_µ_ (confirmed at low speed with independent scanning MFS experiments or made null as described in the following section) are fitting parameters in our study, which aims at extracting and understanding the sole friction coefficient β.

### Drops with Zero Contact-Line Friction.

We first discuss the friction of a drop slightly levitating above a flat solid. Here, we were inspired by recent work reporting a reduced adhesion of carbonated water on hydrophobic and superhydrophobic surfaces (26, 27, 38), where it was proposed that the main resistance to motion arises in the thin CO_2_ gas film supporting the drops (26), whose viscosity $\eta_{g}$ is 15 µPa.s (39). In our work, MilliQ water was carbonated using a commercial SodaStream system, and a drop of sparkling water was placed onto the micropipette just above a transparent superhydrophobic Glaco-treated substrate (see *Materials and Methods* for all experimental details). After gently pushing on the micropipette to the side, the drop-micropipette started to oscillate (Fig. 2 *A* and *B* and Movie S1). Experiments were performed with drops from 1 to 10 µL and slightly different CO_2_ concentrations (rendering different levitation times, see *Materials and Methods*), and the center-of-mass drop oscillations were fit with the ODT model. In all experiments, the fit gave values on the order of $F_{\mu }\approx 10^{-20}\;N$ with errors on the order of $\Delta F_{\mu }\approx 10^{-21}\;N$. However, this value reflects more the properties of the ODT method rather than the friction force for the levitating carbonated drops with zero friction force. Based on the validation data of ODT, such extremely low values reflect real values of $F_{\mu }\ll 1\;\mathrm{nN}$ (see *SI Appendix*, Supplementary Text for more details). We estimate the contact-line friction force to be zero in this case.

**Fig. 3.**
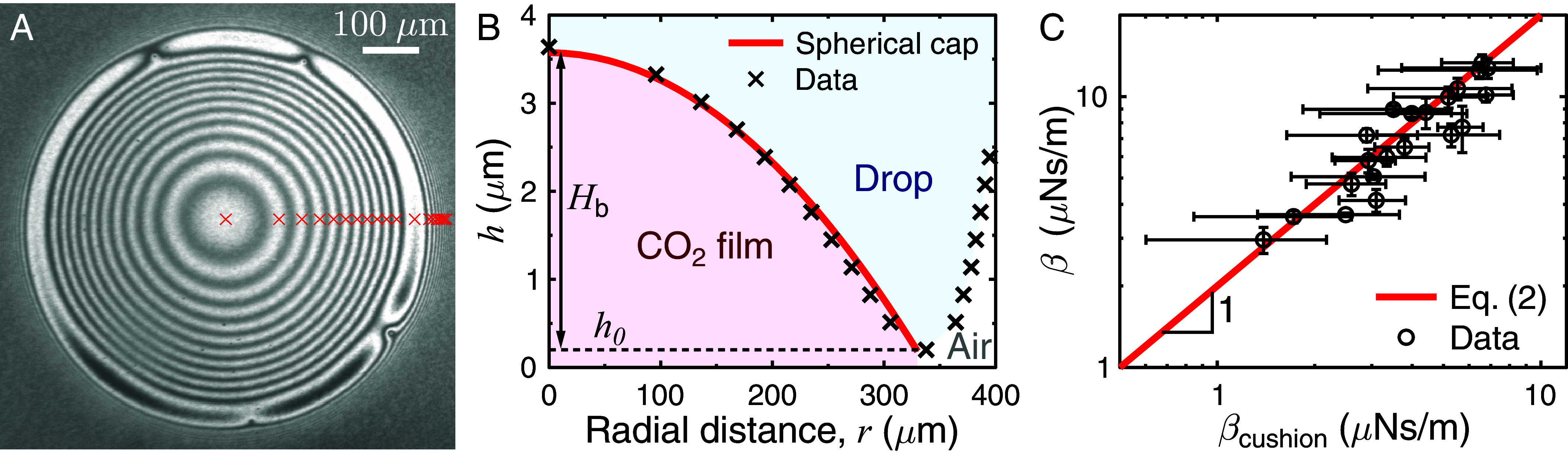
Friction of levitating carbonated water drops on a transparent repellent substrate. (*A*) The contact region between the drop and the substrate as imaged from below with RICM. Each subsequent bright Newton ring (marked with red crosses) shows a change of λ/2 in the thickness of the CO_2_ film, where λ is the wavelength of the LED. (*B*) The gas film height *h* as a function of radial distance *r* from the center of the contact region in (*A*). The CO_2_ blister is a spherical cap (red solid line) with a maximum height $H_{b}$ ≈ 3.5 µm placed on a film with a height *h*_0_ ≈ 0.2 µm. (*C*) Damping coefficient β of the moving drop obtained from oscillating MFS combined with ODT and plotted as a function of the theoretical damping coefficient $\beta_{cushion}$ given by Eq. **2**. The data collapse onto a solid line of $\beta \approx {2\beta }_{cushion}$ (slope of 1 in graph due to log–log axes). The error bars are the 95% CI of the ODT fit for β and the error propagation for the components (and their SD) of Eq. **2** for $\beta_{cushion}.$

To understand the damping in this system, the oscillating MFS experiments were performed together with reflection interference contrast microscopy (RICM) (28–30, 40) from below (see *Materials and Methods* for details). The RICM gave time-resolved images of the contact region between water and its substrate with interference fringes indicating the shape and thickness of the gas film (Fig. 3*A* and Movie S2). By analyzing the position of these Newton rings (crosses in Fig. 3*A*, see *Materials and Methods*), the CO_2_ film profile can be plotted as a function of radial distance *r* (Fig. 3*B*). The Newton rings are symmetrically centered around the contact region center (Movie S2), and the data are well described by a spherical cap on top of a very thin film. Hence, the thickness of this gas “blister” can be written $h=H_{b}-r^{2}/2R_{b}+h_{0}$, where $H_{b}$ is the height of the blister, $h_{0}$ the thickness of the gas film at the perimeter of the contact region, and $R_{b}=\left(l^{2}+H_{b}^{2}\right)/2H_{b}$ is the radius of curvature of the spherical cap. We deduce the viscous damping coefficient of this cap-shaped film:

[2] $$\begin{array}{c} \beta_{\text{cushion}}\approx \eta_{g}\int_{0}^{l}\frac{dA}{h\left(r\right)}\approx -2\pi \eta_{g}R_{b}\text{ln}\left|\frac{l^{2}}{2\left(h_{0}+H_{b}\right)R_{b}}-1\right|\\ \;\approx 2\pi \eta_{g}\frac{l^{2}}{H_{b}}\text{ln}\frac{l}{H_{b}}, \end{array}$$

where we take advantage of the hierarchy in the distances ($h_{0}\ll H_{b}\ll l\ll R_{b}$). The damping coefficients of the carbonated water drops (measured with MFS combined with ODT) from all experiments are plotted in Fig. 3*C* as a function of the value expected from Eq. **2** and deduced from RICM. Data collapse onto a line with a slope of one. The micropipette stiffness and LED wavelength do not affect the scaling (*SI Appendix*, Fig. S4). This experiment thus confirms that the friction of these levitating drops is dominated by the viscous friction in the gas that supports them.

### Drops with Vanishing Contact-Line Friction.

**Fig. 4.**
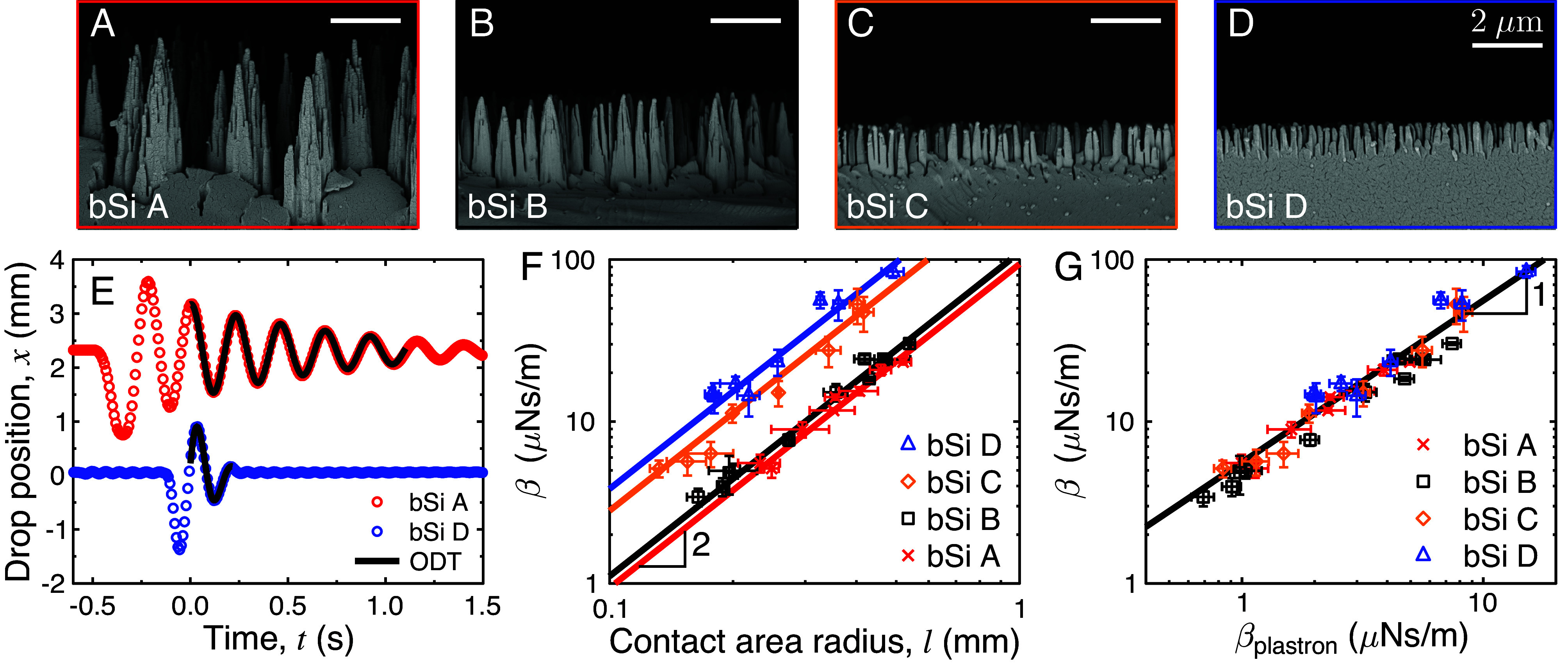
Friction of water drops on superhydrophobic black silicon (bSi). (*A*–*D*) Side-view SEM images of the black silicon samples. All scale bars show 2 μm. (*E*) Drop position as a function of time on bSi A (red data) and D (blue data) for similar water drops (R=0.81±0.07 mm, l=220±20 μm) displaced by about 1.5 mm by a glass fiber contacting them. Oscillations are much more damped on bSi D with shorter texture. Solid black lines show the ODT fits. (*F*) Damping coefficient from oscillating MFS experiments as a function of the contact radius *l* for differently sized water drops on the four bSi samples. Solid lines all show a scaling in *l*^2^. The error bars for β are the 95% CI for the ODT fit and the error for the contact radius is the SD from the time averages of *l*. (*G*) Same data as a function of the theoretical viscous damping coefficient in the plastron β_plastron_ = πη_a_*l*^2^/*H*. The data collapse onto a solid line of β ≈ 5β_plastron_ (slope of 1 in graph due to log–log axes). The error bars are the 95% CI of the ODT fit for β and the error propagation for the components (and their SD) of β_plastron_.

We now discuss whether these results can be generalized to the case of repellent slippery surfaces such as black silicon, where a velocity-dependent force was found to add to and even dominate the line friction (Fig. 1*C*). We used oscillating MFS to measure the damping coefficient on four different black silicon samples (Fig. 4 *A*–*D*), the same surfaces as studied with scanning MFS in ref. 9, see *Materials and Methods*. All samples exhibit conical microstructures, with heights *H* varying from 1 to 4 µm. Looking at the damped oscillations of water drops with *R* ≈ 0.8 mm on samples bSi A and D shows that friction markedly depends on the sample: Oscillations are damped in ~0.25 s on bSi D instead of ~1 s on bSi A (Fig. 4*E*). After using ODT to successfully fit the data, we extract the damping coefficients for differently sized drops (volumes between 1 and 9 µL, allowing us to vary the contact area radius *l*) on the four samples. As seen in Fig. 4*F*, the coefficient β strongly varies with both the black silicon sample and the contact area radius *l*, by a factor 30 between the smallest *l* on sample bSi B to the largest *l* on sample bSi D. Furthermore, this logarithmic representation suggests that data can be described by a quadratic law ($\beta ∼l^{2}$) for all samples, which implies proportionality between friction and contact surface area.

We can compare these observations with the behaviors expected from the various sources of friction discussed earlier. On the one hand, the contact-line friction $F_{\mu }$ is sensitive to the perimeter of the contact and thus scales as *l* (*SI Appendix*, Fig. S5), with no dependency on velocity at low speed, which makes it essential when drops are moving slowly (Fig. 1*C*). In contrast, the Scriven force in the wedge of air bounding the drop is velocity dependent, but it also scales as the contact perimeter, unlike data in Fig. 4*F*. On the other hand (in the liquid), the viscous friction in the drop is velocity dependent, but it scales as *l*^3/2^, that is, with an exponent smaller than observed in Fig. 4*F*. The picture is very different at large viscosity η, as confirmed in *SI Appendix*, Fig. S6 for glycerol on bSi A. Then, the viscous friction *F*_η_ ~ η*Vl*^3/2^/l_c_^1/2^ is found to become dominant at speeds larger than 0.2 mm/s. Other mechanisms (aerodynamic resistance, viscous friction in air) are global, at the scale of the drop, and thus not sensitive to the contact area radius *l*. Hence, none of these forces agree with a friction scaling as *l*^2^; in addition, and consistently, the expected magnitudes of these forces are at least ten times smaller than measured.

Hence, we are left with the plastron friction. It scales as ${(\eta }_{a}V/H)$ *πl*^2^, that is, as *V* and *l*^2^, as respectively observed in Figs. 1*C* and 4*F* and its magnitude is around 10 nN, agreeing again with the data. Furthermore, the phenomenon is general: First, the same β ~ *l*^2^ scaling has been reported for magnetic water drops oscillating on a very slippery superhydrophobic copper surface (33); second, the inverse of the damping time (1/τ ~ β) was also shown to scale linearly with the contact area *l*^2^ of oscillating magnetic drops on four different commercial superhydrophobic coatings (Neverwet, WX2100, Ultra-Ever Dry, and Hydrobead) (41).

The damping coefficient β_plastron_ ~ πη_a_*l*^2^/*H* is expected to be sensitive to the texture design, through its height *H*. This prediction qualitatively agrees with Fig. 4*F* where β, at fixed *l*, decreases when increasing *H*, that is, when going from sample bSi D to sample bSi A. We can further examine this dependency, after evaluating the average thickness *H* of the black silicon microstructure (*SI Appendix*, Table S1) from side-view scanning electron microscopy (SEM) imaging (Fig. 4 *A*–*D*, see *Materials and Methods*). This allows us to calculate the expected damping coefficient β_plastron_ with the air viscosity η_a_ = 18 µPa.s (38). When plotting the results of Fig. 4*F* as a function of β_plastron_ (Fig. 4*G*), all data collapse onto a line with a slope of 1. Hence, we could separately verify the scaling in $l^{2}$ (Fig. 4*F*), $H^{-1}$ (Fig. 4*G*), and V (Fig. 1*C*) expected from the model, which confirms that the resistance opposing the motion of drops on black silicon at intermediate velocity mainly arises from the shearing of the plastron that cannot be considered a purely passive support (42). This scenario is verified for both levitating drops and deposited ones, with differences in the geometry: levitating droplets have a cap-shaped cushion, while droplets on black silicon have a “flat” plastron trapped within the microstructured coating. Other than this, the damping mechanism is the same.

**Fig. 5.**
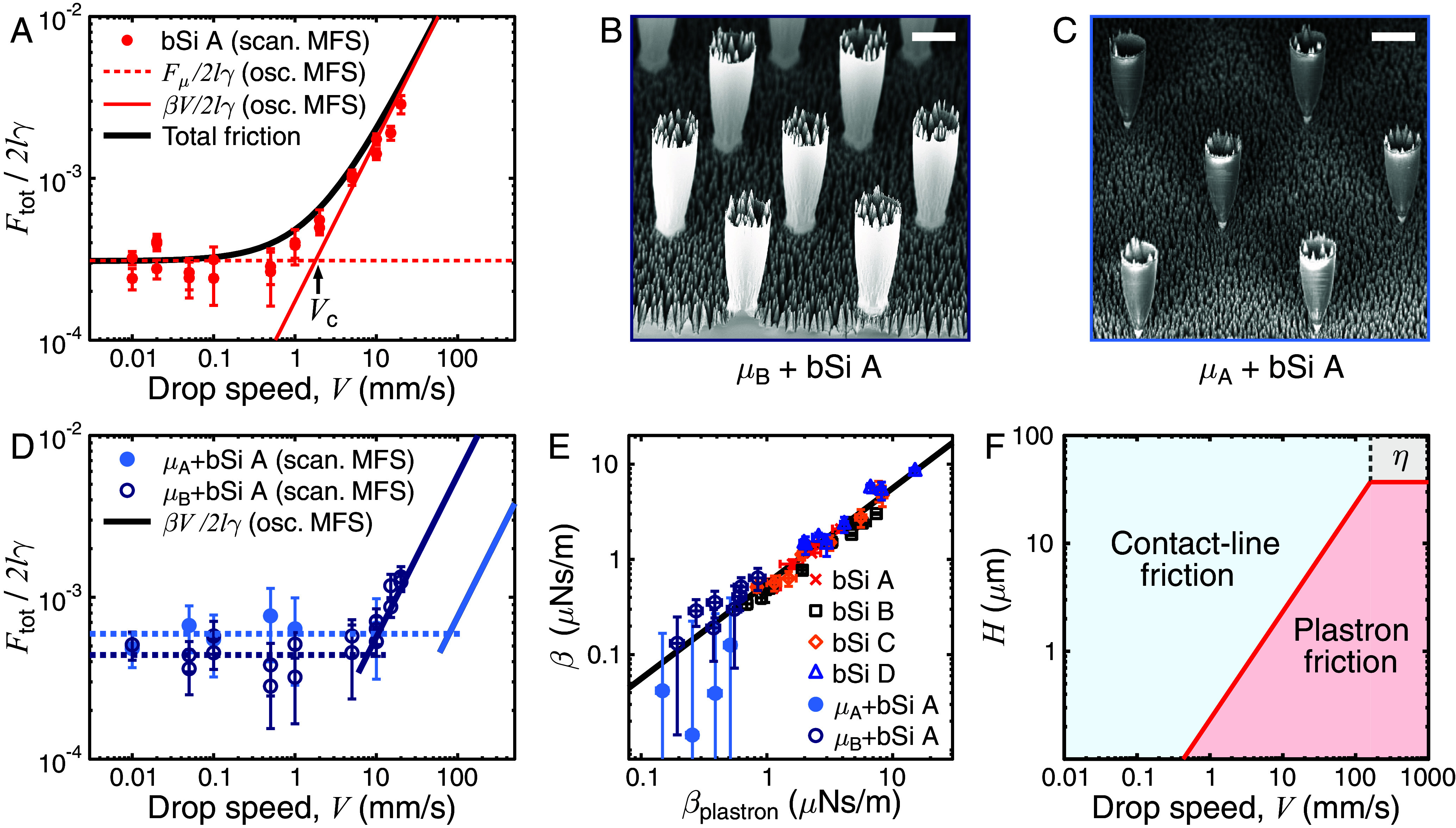
Friction of water drops on the most slippery samples. (*A*) The dimensionless total friction $F_{tot}/2l\gamma$ as a function of the drop speed, as measured by scanning MFS on bSi A (red markers, R=0.92±0.05 mm and l=270±30 μm). Red lines show the average β V/2lγ and $F_{\mu }/2l\gamma$ from the oscillating MFS experiments (Fig. 4) on the same sample, and the black line shows ${(F}_{\mu }+\beta V)/2l\gamma$. (*B* and *C*) SEM image at 45-degree tilt of the µ_B_+bSi and µ_A_+bSi samples. Pillars are less dense on the µ_A_ sample and microtexture on both micropillared samples is bSi A (Fig. 4*A*). The bar shows 10 μm. (*D*) Scanning MFS-measured $F_{tot}/2l\gamma$ vs. *V* on µ_B_+bSi (l=280±30 μm) and µ_A_+bSi (l=290±30 μm). Solid lines are the results from oscillating MFS, and dashed lines are averages from low-speed scanning MFS. (*E*) The damping coefficient β from oscillating MFS experiments as a function of the theoretical plastron damping coefficient $\beta_{plastron}∼\eta_{a}\pi l^{2}\left[\phi_{s}/H+\left(1-\phi_{s}\right)/H_{\mu }\right]$ on bSi and µ+bSi samples. All data collapse onto a line of slope 1. The error bars are the 95% CI of the ODT fit for β and the error propagation for the components (and their SD) of $\beta_{plastron}.$ (*F*) The dominating friction as a function of plastron thickness and drop speed for a water drop with *R* = 1 mm and *l* = 0.2 mm on a surface with $F_{\mu }=10$ nN. The η denotes the region where viscosity in the drop dominates. Error bars in *A*, *D*, and *E* are error propagations of the SD.

As a final demonstration, we compare the scanning MFS experiments at different drop speeds on the most slippery bSi A sample (Fig. 1*C*) with the results from the oscillating MFS experiments (Fig. 5*A*). The averaged quantitative results $F_{\mu }/2l\gamma$ and β V/2lγ from the oscillating MFS are plotted as lines in the graph—these lines are not fits to the scanning MFS data. The dimensionless contact-line friction force measured with scanning MFS in the low drop speed regime is thus in excellent agreement with $F_{\mu }/2l\gamma$ measured with oscillating MFS on the same sample. Furthermore, the two techniques indicate the same onset and scaling of the viscous friction in the plastron. Finally, the total dissipative force $F_{tot}=F_{\mu }+F_{cushion}$ is plotted in Fig. 5*A* as a solid black line where it fits convincingly the data. Hence, our two experimental techniques clearly evidence a previously disregarded friction mechanism on highly slippery materials, that occurring in the plastron.

#### The Plastron Thickness as a Design Parameter for Minimizing Drop Friction Forces.

In order to minimize plastron friction on ultraslippery repellent surfaces, we introduce here a criterion for their design: The plastron must both be thick and maintain a low contact angle hysteresis. One option for such a design is a dual-scale roughness, for example by etching the slippery black silicon bSi A onto micropillars with height $H_{\mu }\approx 44\;\mu m\gg H$ (Fig. 5 *B* and *C*). The samples “µ_A_+bSi” and “µ_B_+bSi” have micropillars (samples µ_A_ and µ_B_) with different densities (or Cassie fraction) $\phi_{s}^{A}=0.04\pm 0.02$ and $\phi_{s}^{B}=0.13\pm 0.06$, as measured from top-view SEM images (*SI Appendix*, Fig. S3 and *Materials and Methods*). Similar samples have been made before (43), but the design process here is unique: Our aim is not to reduce the Cassie fraction but to keep it low while increasing the plastron depth. The dimensionless total friction was measured with scanning MFS on these samples (Fig. 5*D*) and the contact-line friction remains comparable to that on bSi A at low *V*. However, the plastron friction on these samples occurs both in the air between the micropillars and between the bSi cones (with height H measured with side-view SEM, see *SI Appendix*, Fig. S7 and Table S1). This provides $\beta_{plastron}∼\eta_{a}\pi l^{2}\left[\phi_{s}/H+\left(1-\phi_{s}\right)/H_{\mu }\right]∼\eta_{a}\pi l^{2}\phi_{s}/H$ (Fig. 5*E*), i.e., reduced by a factor $\phi_{s}$ compared to bSi samples in Fig. 4. The damping coefficients on these samples were measured using oscillating MFS and they are found to be substantially lower than that on all black silicon samples (*SI Appendix*, Fig. S8), as expected from our arguments. As shown in Fig. 5*D*, the results from the oscillating MFS i) confirm the transition to viscous damping on µ_B_+bSi, and ii) predict the transition on µ_A_+bSi, where scanning MFS could not be used, due to the short size of the sample and the high transition speed. The use of oscillating MFS was thus crucial to properly characterize friction on these samples.

By using this sample design, it is possible to inhibit plastron friction up to $V_{c}\approx 10\;\mathrm{mm}/s$ on µ_B_+bSi and up to $V_{c}\approx 100\;\mathrm{mm}/s$ on µ_A_+bSi compared to $V_{c}\approx 1\;\mathrm{mm}/s$ on the bSi A sample. The transition from a dominating $F_{\mu }$ to $F_{plastron}$ occurs at a plastron thickness of $H_{\mu \to plastron}∼V\eta_{a}\pi l^{2}/F_{\mu }$, whereas the transition from a dominating $F_{plastron}$ to $F_{\eta }$ occurs at $H_{plastron\to \eta }∼2R\eta_{a}/\eta$ independent of the velocity. A transition between $F_{\mu }$ to $F_{\eta }$ occurs at a critical speed $V_{\mu \to \eta }∼2RF_{\mu }/\eta \pi l^{2}$. These equations can be used to design a superslippery sample with minimized friction even at high speed. For example, for a surface with $F_{\mu }=10$ nN, the plastron should be thicker than H≈40 μm to avoid plastron friction (Fig. 5*F*, see *SI Appendix*, Fig. S9 for examples of $F_{\mu }=1,\;100,\;\text{and}\;1,000$ nN surfaces), keeping in mind that this friction is relevant for low viscosity drops (η≪100 mPa.s, *SI Appendix*, Fig. S10).

## Discussion

There is a strong focus on developing extremely slippery repellent surfaces for applications in drag reduction, antifogging, and antiwetting, to name a few. Low friction is attained on superhydrophobic surfaces by reducing the contact made by the drop with the inherently hydrophobic surface. This is achieved through the trapping of an air film (plastron) between the microstructures of the surface, allowing the drop to glide on a cushion of air. Here, we report a plastron friction force on such a slippery repellent surface, as revealed by a MFS in conventional scanning mode as well as in an oscillating mode. We measure the contact-line friction and damping of water drops moving in regimes of vanishing and even zero contact-line friction. We show the importance of the viscous resistance in the seemingly passive air plastron. This force can become dominant for drops with low viscosity (η≪100 mPa.s) moving at 1 to 10 mm/s (a range that depends on the drop size and plastron thickness) on “ultraslippery” materials where line friction $F_{\mu }$ is on the order of 10 nN. The plastron friction increases linearly with the drop speed so that it can become at higher velocity (10 cm/s to 1 m/s) comparable to the line friction of a not-so-slippery repellent sample made of micropillars. This effect thus cannot be neglected in the design of future ultraslippery surfaces. For slightly less slippery samples ($F_{\mu }$ around 300 nN), such as bSi C, superhydrophobic copper surfaces (33), and commercial Glaco and Ultra-Ever Dry samples (18), the transition to the plastron dissipative force-dominated regime occurs at drop speeds in the range of 10 to 100 mm/s, still well below the typical terminal velocity on superhydrophobic surfaces (~m/s).

The current understanding of superhydrophobic sample design has been that reducing the length scale of microstructure (nanograss or nanocones) and diluting it strongly reduces friction. Our work shows that this rule breaks down when plastrons are too thin (see critical plastron thickness values illustrated in Fig. 5*F* and *SI Appendix*, Figs. S9 and S10). Then, friction can drastically increase despite the slippery character of the material. We carefully considered and excluded other velocity-dependent dissipation mechanisms and evidenced the situations where friction mainly originates from the plastron. For a millimetric water drop moving on samples with microscopic structures (H≈1 μm) and a vanishing contact-line friction force $(F_{\mu }<100\;\mathrm{nN}$), the plastron dissipative force becomes key at drop speeds V<10 mm/s. For the special case of a zero contact-line friction, as demonstrated here with levitating carbonated water drops, plastron friction is dominant at most drop speeds. We have found a total of six different highly slippery materials, including well-known commercially available coatings, that all show indications of leading plastron friction. The phenomenon is general, and it needs to be considered in future research and applications. We use our findings to introduce an additional design principle that minimizes contact-line friction and simultaneously reduces the plastron friction, which achieves highly slippery samples at all drop speeds. Our work thus provides the means to design more performant ultraslippery samples in the future.

## Materials and Methods

### Scanning MFS Measurements.

Scanning MFS experiments were performed according to a previously developed protocol (9). Micropipettes were pulled from thick glass capillaries (i.d./o.d. = 0.75 mm/1 mm, World Precision Instruments, model no. TW100-6) using a micropipette puller (Narishige, model no. PN-31). The straight cantilever was cut to a desired length using a microforge (Narishige, model no. MF-900) and calibrated (spring constant $k_{p}$) with a small water droplet as control weight (19). The force-calibrated micropipette was then mounted vertically above the substrate, which rested on a motorized *xyz*-stage. The entire setup was mounted on an active antivibration table (Halcyonics_i4large, Accurion) and shielded by a cardboard box to damp out noise. For the experiments in Fig. 1*B*, a milliQ water drop was placed onto the end of the micropipette so that it rested on the black silicon surface. We have not detected an effect in the force measurement depending on the *z*-position of the micropipette in the drop but tended to perform experiments with the micropipette tip close to the center of the drop. First, the motor was moved in the *y*-direction (out of the field of view) to place the drop-micropipette in the equilibrium *x*-direction. A side-view camera (Canon 90D capturing at 60 to 120 fps using a Canon MP-E macro lens at ~4X magnification) was started to capture the equilibrium position of the pipette (F=0 N); after 3 to 4 s the motor started moving the substrate (V=0.01-20 mm/s with a relative error of 8% for the speed of the stage). The micropipette deflection ($x_{p}$, see Fig. 1*B*) was analyzed using MATLAB (see ref. 19) so that the friction force ($F=k_{p}x_{p}$) could be plotted as a function of time (*SI Appendix*, Fig. S2). The friction force was finally measured in the regime of uniform sliding. A micropipette with spring constant $k_{p}=2.48\pm 0.06\;\text{nN}/\mu m$ was used in the experiments on bSi A as well as for the µ_A_+bSi A and µ_B_+bSi A samples. The upper velocity limit in the experiments was set by the rather short length of the sample (~3 cm). On conventional micropillared samples, micropipettes with $k_{p}=203\pm 2\;\text{and}\;350\pm 20\;\text{nN}/\mu m$ were used.

### Oscillating MFS Measurements.

Three micropipettes were manufactured and calibrated (*k*_p_ = 2.48 ± 0.06, 7.7 ± 0.2 and 10.8 ± 0.4 nN/µm, as described above. The milliQ or carbonated milliQ water drop was placed onto the micropipette as above and the camera was started (at 120 fps). Instead of moving the substrate, the micropipette was gently pushed in the *x*-direction so that the drop started oscillating back and forth (Fig. 2 *A* and *B*). This was repeated 2 to 5 times during one experiment to get at least one measurement where oscillations mostly occurred in the *x*-direction since circular or diagonal oscillations could not be analyzed with ODT. The center-of-mass drop position was analyzed, and the ODT model (see below) was fit to the data using home-written MATLAB codes.

### RICM.

Interference patterns originating from the thin gas film between the drop and transparent Glaco superhydrophobic substrate were probed with a homemade setup. A video microscopy unit (#89-621, Mitutoyo) was coupled with M Plan Apo 2x and 5x objectives (#378-801-6, #378-802-6, Mitutoyo), a USB camera (#MC050MG-SY-UB, Ximea capturing at frame rates of 20 to 30 fps), and a liquid light guide with a 5 mm core diameter (Olympus). A collimation adapter (#SM1U25-A, Thorlabs) focused the light onto the other end of the light guide, and two LED wavelengths were used (λ=455 and 625 nm: #M455L2, #M625L4, Thorlabs). The mounting of both the imaging and illumination units, as well as connecting the light guide to the units, was done with optomechanical components (Thorlabs). The RICM experiment was performed with carbonated drops on Glaco simultaneously with the side-view oscillating MFS experiments (see above). To synchronize the two cameras, the LED light was first turned off; then, both the side- and bottom-view cameras were started, and the LED was turned on. A drop was quickly placed onto the micropipette and pushed to make it oscillate. When the CO_2_ film collapsed (see the end of Movie S3), the LED was turned off and both the cameras were stopped.

### RICM Image Analysis.

Using a home-written MATLAB code, the Newton rings from the RICM experiments were counted as a function of radial distance from the contact region center. Knowing that each subsequent ring corresponds to a change in CO_2_ thickness of λ/2, where λ is the wavelength of the LED (26), the change in film height could be determined. To quantify the absolute thickness of the gas film, we followed the procedure by Panchanathan et al. (26), where the first frame showing the collapse of the gas film was used as a reference for $h_{0}=0$. By playing the video backward and counting the appearance/disappearance of fringes, the levitation thickness $h_{0}$ could be determined. The resulting height profile of the gas film (see example in Fig. 3*B*) was plotted as a function of time during the same time range as analyzed with ODT in the side-view MFS experiments. A spherical cap function was fit to the data, and the time-averaged $h_{0}$, $H_{b}$, and l were used to calculate the damping coefficient in Eq. **2**.

### ODT analysis and simulations.

ODT was done based on the model of oscillating droplet tribometer (33–35, 37). The same model can be used since the forces in our system are similar. The solution of the general harmonic oscillator with viscous and contact-line friction is a piecewise solution for each half-oscillation *n* as

$$x\left(t\right)=x\left(\frac{\mathrm{nT}}{2}+\tau \right)=x_{n+1}\left(\tau \right),$$

where we have:

$$\begin{array}{c} x_{n+1}={\left(-1\right)}^{n}A_{0}\left\{\sqrt{1+\tilde{\beta }}\left[\left(1+\tilde{F}\right)e^{-\tilde{\beta }\pi n}-2\;\tilde{F}\sum_{j=0}^{n}e^{-\tilde{\beta }\pi j}\right]e^{-\tilde{\beta }\omega \tau }\text{cos}\left(\omega \tau -{\text{cos}}^{-1}\left(\frac{1}{\sqrt{1+\tilde{\beta }}}\right)\right)+\tilde{F}\right\}. \end{array}$$

A_0_ is the starting amplitude, ω the angular frequency of the oscillation, $\tilde{F}=F_{\mu }/kA_{0}$ the normalized sliding friction, and $\tilde{\beta }=\beta /\omega m$ the normalized viscous friction coefficient (33, 36). The drop location data were fit to the model based on nonlinear least squares method and interpolating the data to time steps of 1 ms based on smoothing spline using MATLAB. The ODT model was validated for these parameter values by fitting the ODT model to simulated data with similar parameters as the experimental data (*SI Appendix*, Supplementary Text). The simulations were done by solving the drop’s equation of motion forward in time using Runge–Kutta of 4(5) order algorithm using Python package SciPy 1.0 (34, 44, 45). There was good agreement between the used simulation parameters and the parameters obtained from the ODT model, which shows the viability of using the ODT model for oscillating MFS measurements.

### Carbonated Water.

MilliQ water was carbonated using a commercial SodaStream system. A range of different CO_2_ concentrations was used, rendering different levitation times (a few to tens of seconds) of the drop. By measuring the pH of the sparkling water using a micro-pH meter (Mettler Toledo SevenExcellence), the concentrations could be calculated as $C_{0}=10^{-2\cdot pH}/K_{a1}$, where $K_{a1}=4.33\cdot 10^{-7}$ is the first dissociation constant of H_2_CO_3_ (46). Our concentrations ranged between $C_{0}=14\pm 0.6$ mM (pH=4.11) and $C_{0}=44\pm 0.2$ mM (pH=3.86), assuming an error of ΔpH=±0.01 for the pH meter. According to the work of Panchanathan et al. (26), these concentrations should yield levitation times of 3 to 12 s, which corresponds to what we observed.

### Preparation of the Glaco Sample.

Microscope glass slides were thoroughly cleaned by rinsing and scrubbing with cotton sticks in acetone (≥99.5 %, Sigma-Aldrich). Then, they were ultrasonicated for 10 min in acetone (≥99.5 %), followed by rinsing with isopropyl alcohol (≥99.7 %, Sigma-Aldrich). After drying under nitrogen flow, the slides were treated for 5 min with oxygen plasma for homogenization of surface chemistry. Finally, the samples were sprayed with Glaco, following the instructions from the supplier. Both spraying and drying were done on vertically mounted slides. The drying time was 24 h. The advancing and receding contact angles of water on these Glaco samples were $\theta_{adv}=(168\pm {4)}^{\circ }$ and $\theta_{\text{rec}}={\left(166\pm 5\right)}^{\circ }$, confirming that these samples are superhydrophobic. These contact angle measurements were done using conventional contact angle goniometry (Biolin Attension Theta) by growing a 1 µL droplet, bringing it in contact with the surface, growing the drop 1 µL more, and then centering the needle in the backside of the droplet. The growth speed for this process was 0.05 µL/s. Then, the droplet volume was grown for 15 µL more at the rate of 0.05 µL/s while it was recorded, giving the advancing contact angle. For the receding angle, the droplet was grown for 10 µL more with a rate of 0.05 µL/s, and then, it was sucked in for 25 µL. After each receding angle experiment, a volume of 50 µL was dispensed aside to prevent contamination of water, and then, the next advancing angle was measured. The measurement was repeated 10 times giving an average advancing and receding contact angle with their SD.

### Fabrication of Black Silicon Samples.

Black silicon nanopillars were fabricated with a maskless cryogenic deep reactive ion etching process (47). The plasma etcher used was Oxford Plasmalab System 100, Oxford Instruments. For all types of black silicon, the etching temperature −110 °C, the pressure 10 mTorr, and the etching time 7 min were kept constant. To obtain the different black silicon topographies A, B, C, and D, the SF_6_ gas flows (in sccm) were 40, 37.6, 35.3, and 32.9, the O_2_ gas flows (in sccm) were 18, 20.4, 22.8, and 25.1, and the forward powers (in W) were 6, 6, 5, and 4, respectively. The samples were then made superhydrophobic by applying a plasma-enhanced chemical vapor deposited (PECVD) fluoropolymer coating (Oxford Plasmalab 80+, Oxford Instruments). The parameters for this coating were 50 W power, 250 mTorr pressure, 100 sccm of CHF_3,_ and a deposition time of 5 min. The samples bSi B–D were the same as used in ref. 9, whereas bSi A was new for this project but made with the same parameters as described above. Sample bSi E from ref. 9 was not used in this work since water drops would not oscillate enough to render reliable $F_{\mu }$ and β data using ODT.

### Fabrication of the Micropillared Sample.

Micropillar surfaces were fabricated with an anisotropic cryogenic deep reactive ion etching process using an oxide hard mask. The starting point was a silicon wafer with a 510 nm thick thermal oxide. Micropillars were defined by an optical lithography process with AZ-5214E photoresist, using Karl Suss MA-6 mask aligner. An image reversal process with 1 s first exposure and 10 s flood exposure was used due to the polarity of the photomask. The pattern was 10 μm diameter circular micropillars in a hexagonal lattice and a theoretical solid fraction (Cassie fraction) of 2.5% and 7.4% for µ_A_ and µ_B_, respectively. After lithography, reactive ion etching (Oxford Plasmalab 80+, Bristol, UK) was used to etch the silicon dioxide. The etching parameters were 30 W power 200 mTorr pressure, 25 sccm CHF_3_ flow, and 25 sccm Ar flow, and the etching time was 14 min. After etching, the photoresist was stripped. The silicon micropillars were then etched with an anisotropic silicon etch (Oxford Plasmalab System 100, Oxford Instruments). The Si etching parameters were forward power 3 W, ICP power 1,050 W, −110 °C temperature, 8 mtorr pressure, 6 sccm O_2_ flow, and 40 sccm SF_6_ flow. The etching time was 18 min. Afterward, the oxide mask was removed with buffered HF. The depth of the micropillars was measured by a profilometer (Bruker Dektak XT) to be 44 µm. Finally, the same PECVD fluoropolymer coating as for the black silicon samples was deposited on top of the micropillars. SEM images of the final samples are shown in *SI Appendix*, Fig. S3.

### Fabrication of Etched Micropillared Samples.

The samples with black silicon on top of micropillars were fabricated by a combination of the two processes. First, the micropillars were fabricated as described above and coated with PECVD fluoropolymer. These conventional superhydrophobic samples were probed with MFS (black data in Fig. 1*C*). Then, the fluoropolymer coating was removed with oxygen plasma, and the black silicon A process (described above) was applied. As a last step, the same PECVD fluoropolymer coating that was used for both the black silicon and the micropillar samples was deposited on top of the combined micropillars + bSi samples.

### SEM Measurements.

The SEM imaging was done using Zeiss Sigma VP SEM at a low acceleration voltage (1.0 kV) with an in-lens detector. Before imaging, the samples were coated with gold–palladium (Au/Pd) coating with Leica EM ACE600 high-vacuum sputter coater in the following manner. The bSi A (Fig. 4*A*) and µ_B_+bSi A sample (Fig. 5*B*) was coated with 5 nm Au/Pd from the top and 5 nm Au/Pd from the side, while samples bSi B–D were coated with 8 nm Au/Pd from the side. No conductive coating was applied on the samples µ_A_+bSi and µ_B_+bSi shown in *SI Appendix*, Fig. S7. The SEM imaging of micropillared samples µ_A_ and µ_B_ before the black silicon etching was done with 4.0 kV acceleration voltage with an SE2 detector, and no conductive coating was applied on these samples.

## Supplementary Material

Appendix 01 (PDF)

Movie S1. **Side-view movie of an oscillating MFS experiment.** Side-view movie (SuppMov1.mp4) of an oscillating micropipette force sensor experiment with a carbonated water drop (*R* = 1.12 ± 0.03 mm, *l* = 350 ± 1 μm; same as in **Fig. 2a** in the main text) on Glaco. The movie is slowed down 4 times.

Movie S2. **Bottom-view movie of an oscillating MFS experiment.** Bottom-view movie (SuppMov2.mp4) using reflection interference contrast microscopy of the contact region between the carbonated water drop and the Glaco surface (same as in **Fig. 2a** in the main text). The time-range of the movie corresponds to that analysed with oscillating droplet tribology in **Fig. 2a** in the main text.

Movie S3. **Bottom-view movie (long version) of an oscillating MFS experiment.** Bottom-view movie (SuppMov3.mp4) using reflection interference contrast microscopy of the contact region between the carbonated water drop and the Glaco surface from the entire experiment in **Fig. 2a** in the main text, showing the collapse of the CO_2_ film at the end.

## Data Availability

All data needed to evaluate the conclusions in the paper are present in the paper and *SI Appendix*, Tables S2–S16. The raw data files and MATLAB codes are shared on Zenodo (48).
