# Supplementary material for: Toward vanishing droplet friction on repellent surfaces
Source: Proc Natl Acad Sci U S A. 2024 Apr 15;121(17):e2315214121. doi: 10.1073/pnas.2315214121 (PMC11047067; doi:10.1073/pnas.2315214121)
Supplement: Supplementary file 1 — Appendix 01 (PDF) [file pnas.2315214121.sapp.pdf]

Supplementary Materials for

Toward vanishing droplet friction on repellent surfaces

Matilda Backholm,[1,2,*] Tytti Kärki,[1,2,†] Heikki A. Nurmi,[1,2,†] Maja Vuckovac,[1,2] Valtteri Turkki,[1,2] Sakari Lepikko,[1,2] Ville Jokinen,[3] David Quéré,[4] Jaakko V.I. Timonen[1,2] and Robin H.A. Ras[1,2,*]

[1] Department of Applied Physics, Aalto University, 02150 Espoo, Finland
[2] Center of Excellence in Life-Inspired Hybrid Materials, Aalto University, Espoo Finland
[3] Department of Chemistry and Materials Science, Aalto University, 02150 Espoo, Finland
[4] Physique et Mécanique des Milieux Hétérogènes, UMR 7636 du CNRS, PSL Research University, ESPCI, 75005 Paris, France

*E-mail: matilda.backholm@aalto.fi, robin.ras@aalto.fi
†These authors contributed equally.

This PDF file includes:

Supplementary Text "Validation of ODT model for oscillating MFS"
Supplementary Text "Solid fraction on bSi"
Supplementary References
Figs. S1 to S14
Table S1 to S16
Movies S1 to S3

Other Supplementary Materials for this manuscript include the following:

Movies S1 to S3

Supplementary Text "Validation of ODT model for oscillating MFS"

The oscillating droplet tribology (ODT) analysis for oscillating MFS was validated against simulated data, which was generated by solving the general harmonic oscillator with viscous and friction force equation ($m(\mathrm{d}^2x/\mathrm{d}t^2) = -k(x - x_p) - \beta V - F_\mu$) with Runge-Kutta method of 5(4) order algorithm (*1–3*). In these simulations, the used inputs were potential well location x_p, mass of the droplet *m*, spring coefficient *k*, viscous coefficient β and the magnitude of line friction $|F_\mu|$, which were used to simulate the droplet centroid location *x*. The potential location was moved sinusoidally near the resonance frequency of the system ($f = \sqrt{\frac{k}{4m\pi^2}}$, for harmonic oscillator), which caused the simulated drop to move. The ODT model was then fit to the simulated data. This allowed the comparison of the simulation input parameters and the solution of the ODT model (**Fig. S11**), which were calculated for a spring coefficient $k = 2\ \mathrm{mN/m}$, a viscous coefficient $\beta \in [10^{-8}, 10^{-4}]\ \mathrm{Ns/m}$, a line friction $|F_\mu| \in [10^{-9}, 10^{-6}]$ N and a droplet mass *m* between 1 mg and 10 mg.

We carefully analyzed the effect of the aerodynamic dissipative force in our *oscillating* MFS experiments. The drag force from the air on the oscillating micropipette and drop was calculated as $F_\mathrm{air} \sim \rho_\mathrm{a}\mathrm{Re}^{1/2}SV^2$ (*4*), where ρ_a is the density of air, Re is the Reynolds number of the pipette ($\mathrm{Re_p} \sim \rho_\mathrm{a}2R_\mathrm{p}v_\mathrm{p}/\eta_\mathrm{a}$, where R_p is the pipette radius and v_p the pipette speed) or drop ($\mathrm{Re_d} \sim \rho_\mathrm{a}2RV/\eta_\mathrm{a}$), *S* is the reference area of the pipette ($S_\mathrm{p} \sim 2R_\mathrm{p}L_\mathrm{p}$, where L_p is the cantilever length) or drop ($S_\mathrm{d} \sim \pi R^2$). We calculated the sum of the air resistance on the pipette and drop as a function of time from many oscillating MFS experiments with water or carbonated water. However, we found that the effect from this is several orders of magnitude lower than the effect of the viscous dissipation of βv (**Fig. S12**). This finding is in agreement with the work by Mouterde *et al.* (*4*), showing that air resistance becomes relevant at drop speeds of order 1 m/s. The typical max speed of drops in our oscillating MFS experiments is ~10 to 100 mm/s, and air resistance can thus safely be left out of the ODT equation.

The combined error was calculated as $\sqrt{\frac{(F_{\mu,\mathrm{sim}} - F_{\mu,\mathrm{fit}})^2}{F_{\mu,\mathrm{sim}}^2} + \frac{(\beta_\mathrm{sim} - \beta_\mathrm{fit})^2}{\beta_\mathrm{sim}^2}}$, where subscript sim refers to used simulation parameter and fit refers to value from the fitted ODT model. The inaccurate area the lower left corner of **Fig. S11d-f** is due to the poor fitting, which could be improved with adjusting the starting guesses in the fitting. In addition, a longer simulation time could improve the accuracy since the oscillations continue longer than the simulation time (8529 ms for the smaller mass and 9404 ms for larger mass, with 8000 ms simulation time for decaying). The total simulation time is different, since the total time consists of a short waiting at the start (100 ms), movement of the potential based on resonance frequency (varied) and the decaying (8000 ms). Overall, the accurate range of ODT model coincides with the measured parameters in the main experiments, which gives confidence in the measured results and the used model. As the used simulation friction force decreases, the solved friction force decreases faster resulting in larger negative error (**Fig. S11b**). These increasing errors hinder the accurate measurement of vanishing line friction ($F_\mu \ll 1$ nN), which is the case for the levitating carbonated droplets. For the levitating carbonated drop, the friction force is zero as the drop does not touch the surface. However, the analytical fit will always give a non-zero value for the friction force (on the order of $F_\mu = 10^{-20}$N),

even if the value is clearly incorrect. In these cases, the friction force is estimated to be zero instead of the extremely low number, since the accuracy of the low force is uncertain, and estimating the force as zero is more accurate.

The two types of dissipation can be distinguished from each other when the other one is dominating, since the viscous force dominated dissipation leads to clear exponential decay of the oscillations, while the friction force dominated dissipation leads to linear dissipation (**Fig. S13**). While the friction and viscous forces are of similar magnitude, then the dissipation is a linear combination of these two types of decay. This holds true even when the resonance frequency of the system changes, which can be seen by comparing simulated droplet data with the two different masses. The effect of time step interpolation was investigated by fitting the ODT model for the same location data set with different time steps (**Fig. S14**). The different time steps were done by interpolating the original simulated data (1000 fps) to different time steps from 1000 fps to 30 fps using smoothing spline. Some of the data points near 30 fps are not visible in the figures, since the values are vastly off from the used simulation input value such as the smallest friction force value $2.1 \cdot 10^{-19}$ N with 35 fps capture rate. Then this larger time step data was returned to 1000 fps using smoothing spline and the ODT model was fit to the data. The results of these fits show that the 60 fps is needed for good fit, which is lower than the experimentally used 120 fps.

Supplementary Text “Solid fraction on bSi”

The solid fraction ϕ is the relative contact that the liquid makes with the solid under the drop. It is very difficult to measure experimentally on the bSi samples due to their rounded tops, randomness and microscopic sizes. However, we can theoretically estimate the solid fraction from the measured contact-line friction: $\frac{F}{2l\gamma} \sim \phi \log\frac{1}{\phi}$ (*5*). For our four bSi samples (A to D), $\frac{F}{2l\gamma} \approx 3 \cdot 10^{-4}$ to $2 \cdot 10^{-2}$ (*6*), giving $\phi \approx 0.00004 - 0.004$. These ultra-low values are consistent with the ultra-slippery nature of these materials.

Supplementary References

1. M. Junaid, H. A. Nurmi, M. Latikka, M. Vuckovac, R. H. A. Ras, Oscillating droplet tribology for sensitive and reliable wetting characterization of superhydrophobic surfaces. *Droplet* **1**, 38–47 (2022).

2. J. R. Dormand, P. J. Prince, A reconsideration of some embedded Runge—Kutta formulae. *J. Comput. Appl. Math.* **15**, 203–211 (1986).

3. P. Virtanen, R. Gommers, T. E. Oliphant, M. Haberland, T. Reddy, D. Cournapeau, E. Burovski, P. Peterson, W. Weckesser, J. Bright, S. J. van der Walt, M. Brett, J. Wilson, K. J. Millman, N. Mayorov, A. R. J. Nelson, E. Jones, R. Kern, E. Larson, C. J. Carey, İ. Polat, Y. Feng, E. W. Moore, J. VanderPlas, D. Laxalde, J. Perktold, R. Cimrman, I. Henriksen, E. A. Quintero, C. R. Harris, A. M. Archibald, A. H. Ribeiro, F. Pedregosa, P. van Mulbregt, SciPy 1.0 Contributors, A. Vijaykumar, A. P. Bardelli, A. Rothberg, A. Hilboll, A. Kloeckner, A. Scopatz, A. Lee, A. Rokem, C. N. Woods, C. Fulton, C. Masson, C. Häggström, C. Fitzgerald, D. A. Nicholson, D. R. Hagen, D. V. Pasechnik, E. Olivetti, E. Martin, E. Wieser, F. Silva, F. Lenders, F. Wilhelm, G. Young, G. A. Price, G.-L. Ingold, G. E. Allen, G. R. Lee, H. Audren, I. Probst, J. P. Dietrich, J. Silterra, J. T. Webber, J. Slavič, J. Nothman, J. Buchner, J. Kulick, J. L. Schönberger, J. V. de Miranda Cardoso, J. Reimer, J. Harrington, J. L. C. Rodríguez, J. Nunez-Iglesias, J. Kuczynski, K. Tritz, M. Thoma, M. Newville, M. Kümmerer, M. Bolingbroke, M. Tartre, M. Pak, N. J. Smith, N. Nowaczyk, N. Shebanov, O. Pavlyk, P. A. Brodtkorb, P. Lee, R. T. McGibbon, R. Feldbauer, S. Lewis, S. Tygier, S. Sievert, S. Vigna, S. Peterson, S. More, T. Pudlik, T. Oshima, T. J. Pingel, T. P. Robitaille, T. Spura, T. R. Jones, T. Cera, T. Leslie, T. Zito, T. Krauss, U. Upadhyay, Y. O. Halchenko, Y. Vázquez-Baeza, SciPy 1.0: fundamental algorithms for scientific computing in Python. *Nat. Methods* **17**, 261–272 (2020).

4. T. Mouterde, P. S. Raux, C. Clanet, D. Quéré, Superhydrophobic frictions. *Proc. Natl. Acad. Sci. U. S. A.* **116**, 8220–8223 (2019).

5. D. Quéré, Wetting and Roughness. *Annu. Rev. Mater. Res.* **38**, 71–99 (2008).

6. M. Backholm, D. Molpeceres, M. Vuckovac, H. Nurmi, M. J. Hokkanen, V. Jokinen, J. V. I. Timonen, R. H. A. Ras, Water droplet friction and rolling dynamics on superhydrophobic surfaces. *Commun. Mater.* **1**, 64 (2020).

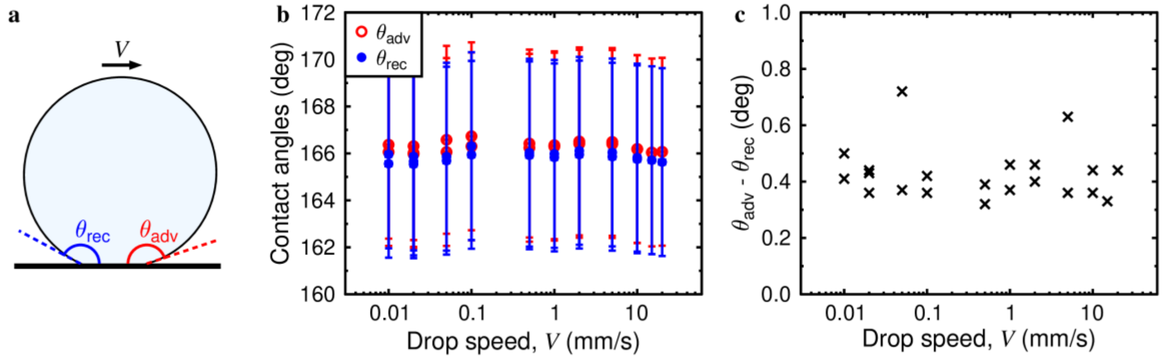

Fig. S1.

Contact angles on bSi A. a) Schematic drawing of the advancing (θ_{adv}) and receding (θ_{rec}) contact angles for a drop moving with a speed V on a black silicon superhydrophobic surface. **b)** The contact angles from the experiments in main **Fig. 1B-C** remain constant for a water drop moving at different speeds on bSi A. The error for the contact angles (± 4 deg) is evaluated from previous simulations for superhydrophobic substrates (*6*). **c)** The contact angle hysteresis (from the data in **b**) remains constant, indicating that there is no viscous effect on the contact angles and that the contact-line friction remains constant within this drop speed range.

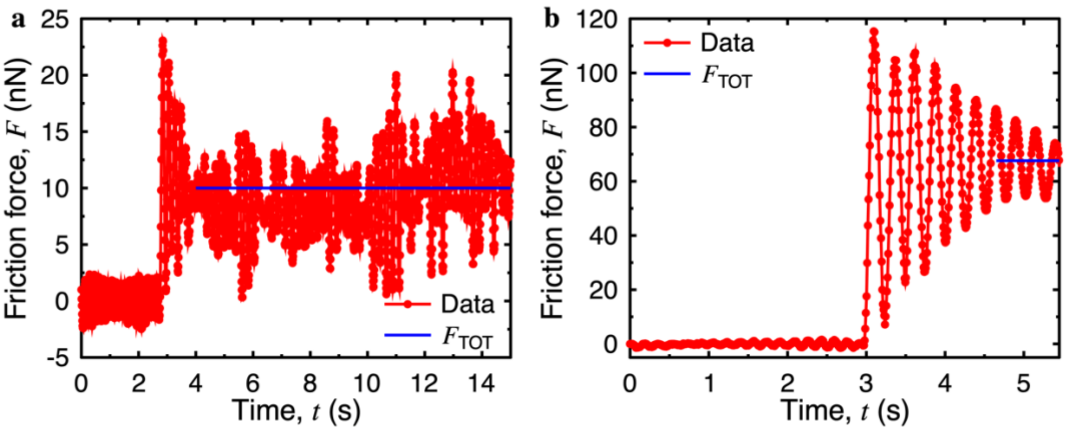

Fig. S2.

Micropipette force sensor experiments on bSi A. Force versus time data from micropipette force sensor (MFS) experiments with water drops on etched black silicon bSi A at **a)** $V = 0.5$ mm/s ($R = 860 \pm 10$ μm, $l = 250 \pm 10$ μm); **b)** $V = 15$ mm/s ($R = 930 \pm 10$ μm, $l = 250 \pm 10$ μm).

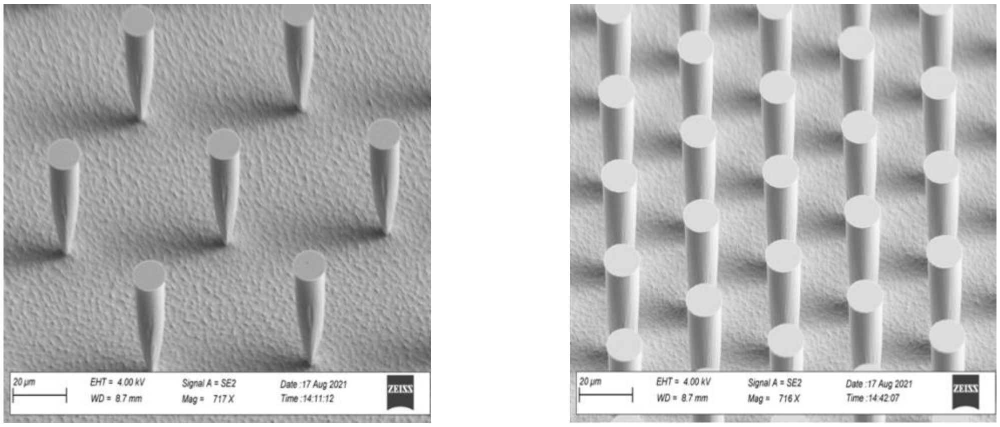

Fig. S3.

Conventional micropillared samples. SEM images (taken at an angle of 45 degrees) of the micropillared samples μ_A (left) and μ_B (right) before etching bSi onto them.

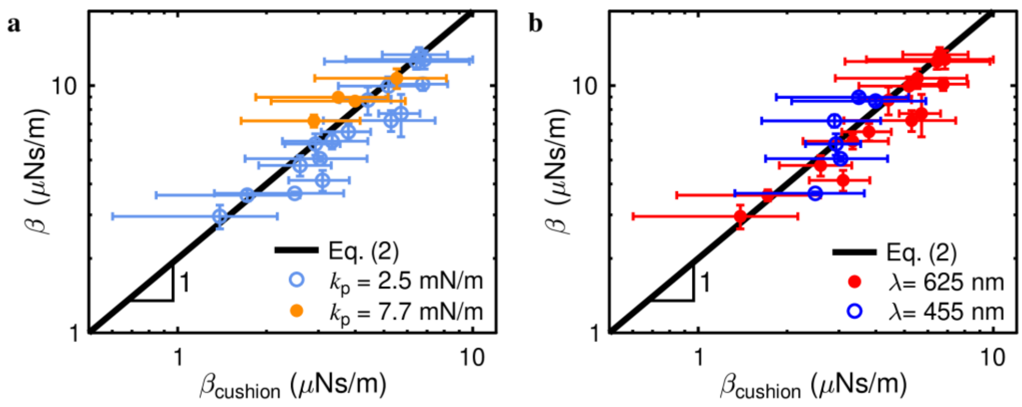

Fig. S4.

Drop damping coefficient for carbonated drops levitating on Glaco. The data in main **Fig. 3C** collapse using **a)** two different micropipette spring constants, as well as **b)** two different wavelengths of the LED.

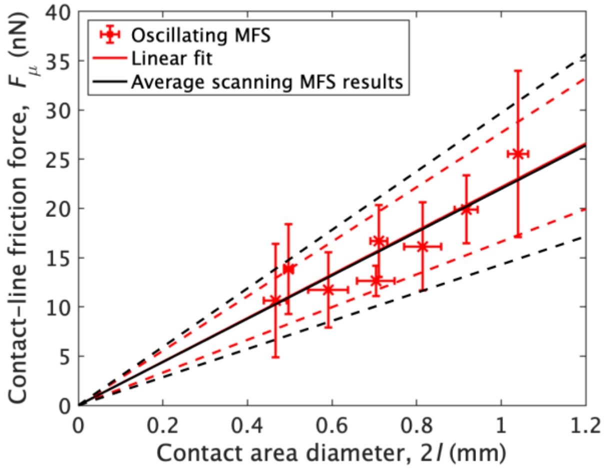

Fig. S5.

Contact-line friction measured with scanning and oscillating MFS on bSi A. The $\boldsymbol{F_\mu}$ from the ODT fit performed on the oscillating MFS data for differently sized water drops (red crosses) are in excellent agreement with the linear fit (solid black line) done to the low-velocity scanning MFS data (data points not plotted for clarity, see data in **Fig. 5A**). The dashed black lines are the error lines for the scanning MFS experiments.

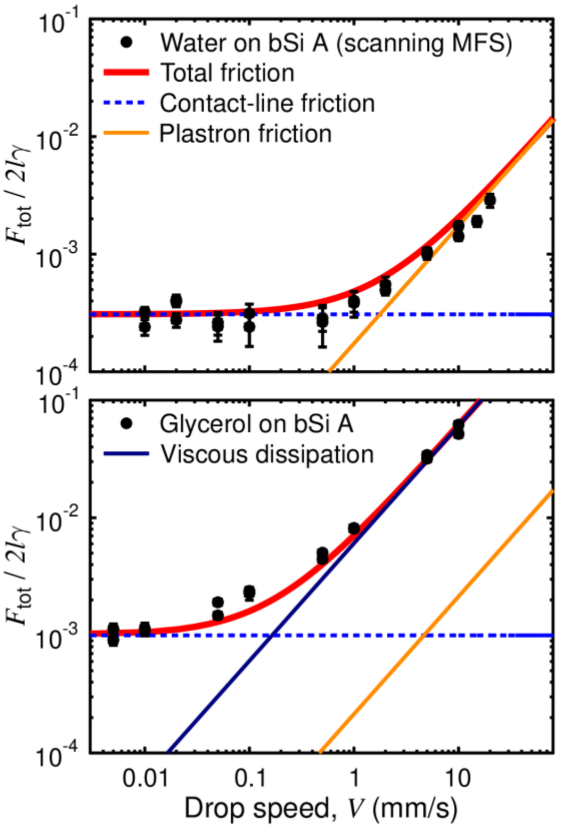

Fig. S6.

Friction of water and glycerol drops on bSi A. The dimensionless total friction measured using scanning MFS with drops of either water (top, $R = 0.92 \pm 0.05$ mm and $l = 270 \pm 30$ μm, same as **Fig. 5A** in main text) or glycerol (bottom, $R = 0.88 \pm 0.01$ mm/s, $l = 290 \pm 10$ μm). Drops are similar, but the behaviours are quite different, since the deviations from the plateau value at low speed appear earlier with glycerol, above 0.2 mm/s instead of 2 mm/s for water. We also note that the plateau value is slightly higher for glycerol with smaller surface tension, thus of larger contact angle hysteresis. When we add to the graph the calculated plastron friction (orange line) and drop viscous friction (dark blue line), the origin of the difference becomes clear. Both the frictions are linear in velocity, but, logically, the viscous friction in the drop (dark line) becomes dominant at much smaller velocity when the liquid viscosity is higher ($\eta_{glycerol} \approx 1500\ \eta_{water}$), so as to dominate the plastron friction (orange line), whatever the velocity. No oscillating MFS experiments could be performed with glycerol drops and we simply assume that the plastron friction (independent of η) is the same as for water. The dashed blue line is a fit to the plateau at low speed, and the solid dark line is not a fit but just the calculated Mahadevan-Pomeau friction $F_\eta \sim V\eta\pi l^2/2R$ with the parameters of the experiment.

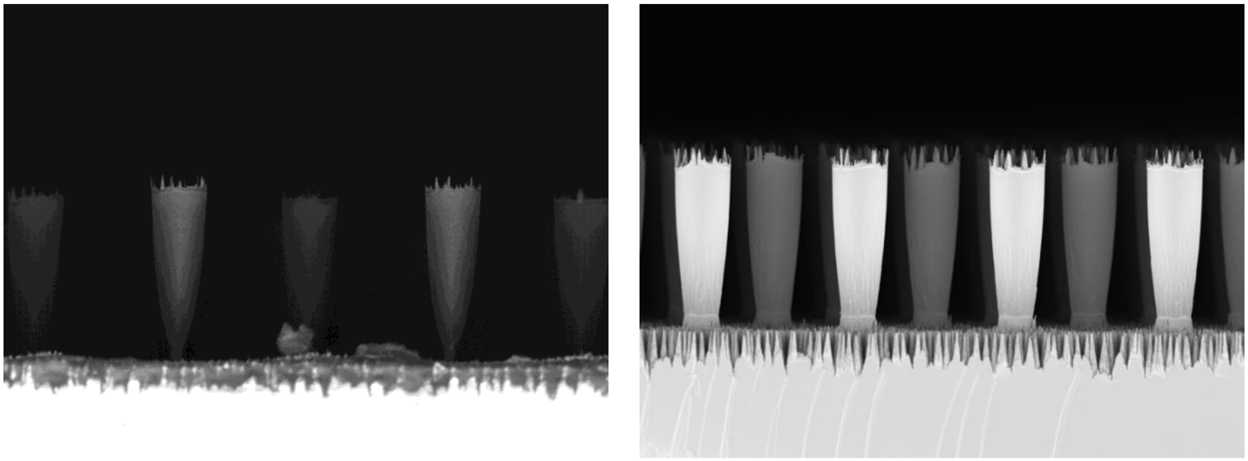

Fig. S7.
Etched micropillared samples. Side-view scanning electron microscopy (SEM) images of μ_A+bSi (left) and μ_B+bSi (right) samples, where the microstructure is bSi A. The top width of the µ-pillars is ~10 µm, which provides the scale.

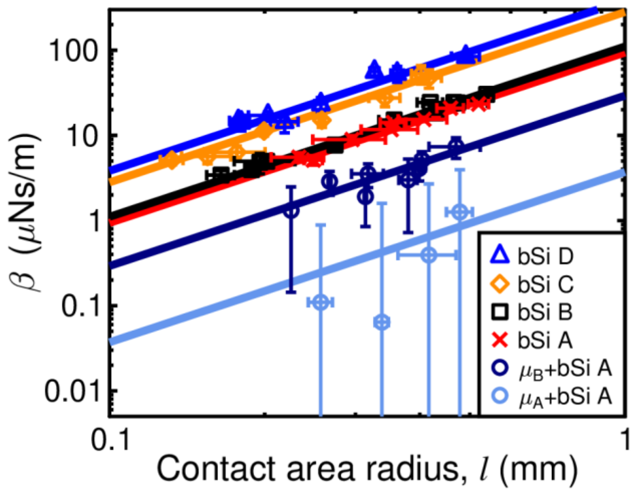

Fig. S8.

Friction of water on black silicon (bSi) and μ+bSi samples. The damping coefficient from oscillating droplet tribology (ODT) as a function of the contact area radius of differently sized drops. The solid lines are fits of $\beta \sim l^2$ to the data. The error bars for β are the 95% confidence intervals for the ODT fit and the error for the contact area radius is the standard deviations from the time-averages of l. Frictions on the etched pillared samples μ + bSi are significantly lower than on normal black silicon bSi.

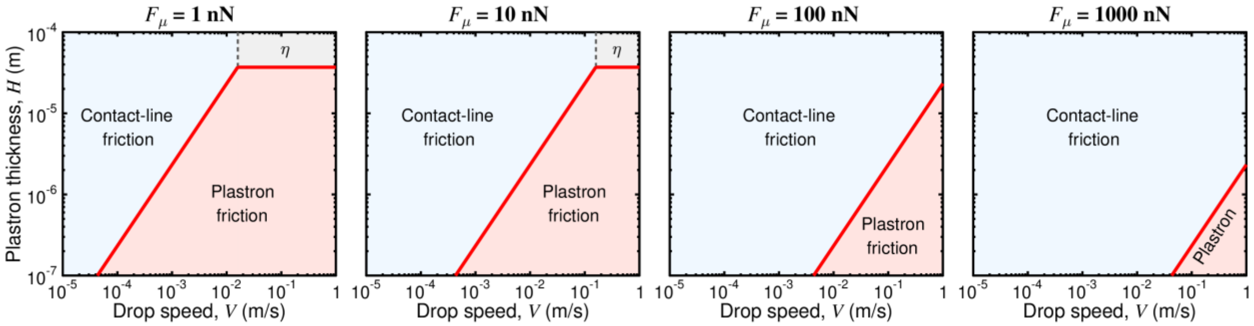

Fig. S9.

Expected regimes of friction for different contact-line frictions. The dominating friction as a function of plastron thickness and drop speed for a $R = 1$ mm water drop (with $l = 0.2$ mm) on surfaces with $F_\mu = 1$, 10, 100 and 1000 nN. The grey area (marked with η) denotes the regime where the viscous dissipation in the drop dominates. The plastron friction is especially relevant on highly-slippery materials (low F_μ), and it appears, at fixed H, above a threshold velocity, as discussed in the main text.

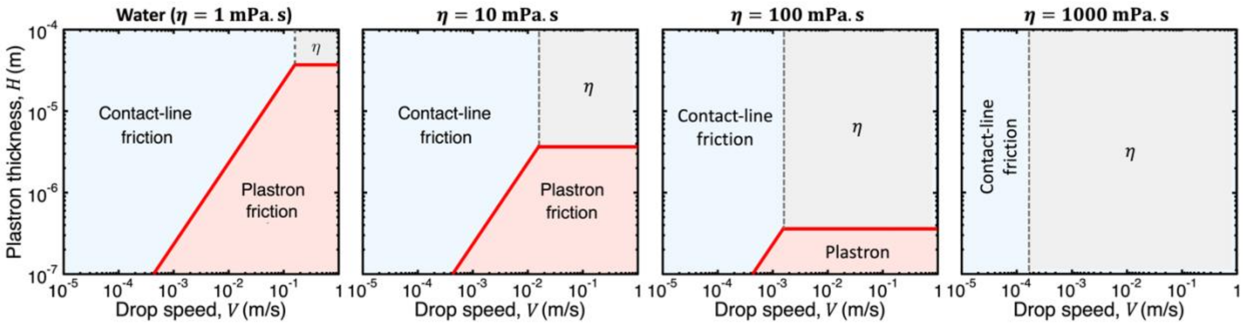

Fig. S10.

Expected regimes of friction for drops with various viscosities. The dominating friction as a function of plastron thickness and drop speed for a $R = 1$ mm drop (with $l = 0.2$ mm) on a $F_{\mu} = 10$ nN surface at different liquid viscosities ($\eta = 1, 10, 100, 1000$ mPa.s). The grey area marked with η denotes the region where viscous dissipation in the drop dominates. The plastron friction appears to be relevant when the drop viscosity is below 10 mPa.s; conversely, bulk viscosity logically imposes the friction for liquids such as glycerol (η ≈ 1000 mPa.s), as seen in **Fig S6**.

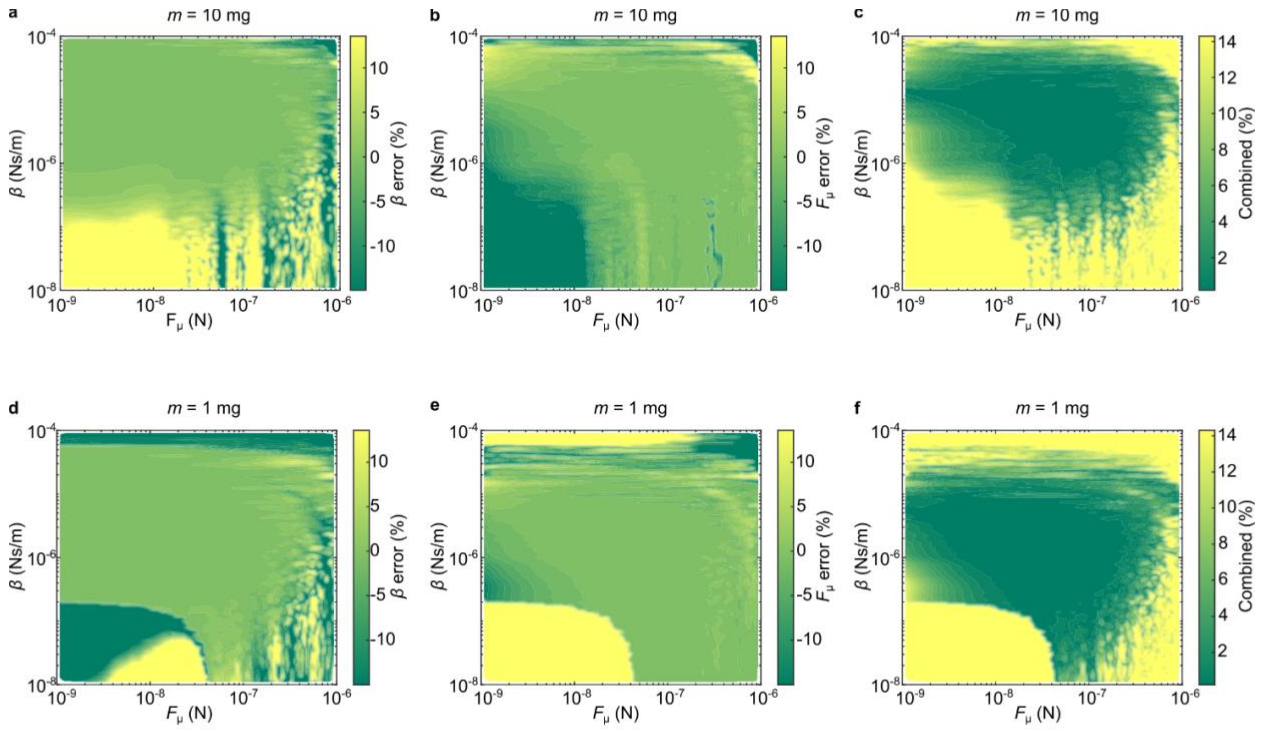

Fig. S11.

Accuracy maps of ODT analysis based on simulated data. The difference between the simulation input value and the solved ODT value is used as estimation on the error in **a**, **b**, **d**, and **e**. Estimation the total accuracy of ODT analysis is calculated by the combined errors of viscous and friction coefficients in **c** and **f**. The error magnitudes larger than 15% are capped to 15% in magnitude for increased readability.

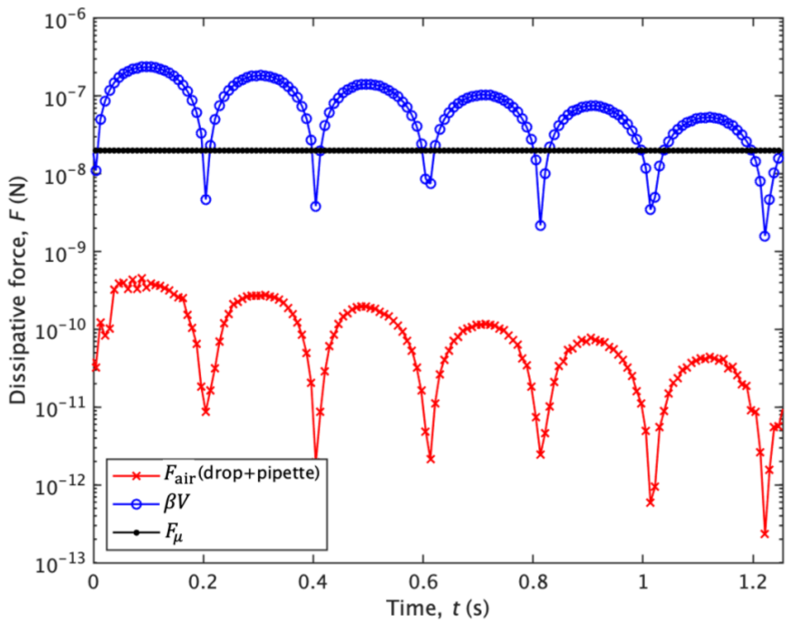

Fig. S12

Friction in oscillating MFS experiments. The total air resistance on the pipette and drop (F_{air}, red), viscous force (βV) from the ODT (blue), and contact-line friction (F_{μ}) from the ODT (black) for a water drop with $R = 1.3$ mm and $l = 0.4$ mm oscillating on bSi A. The air resistance is several orders of magnitude smaller than the other frictions and can thus be neglected in the ODT.

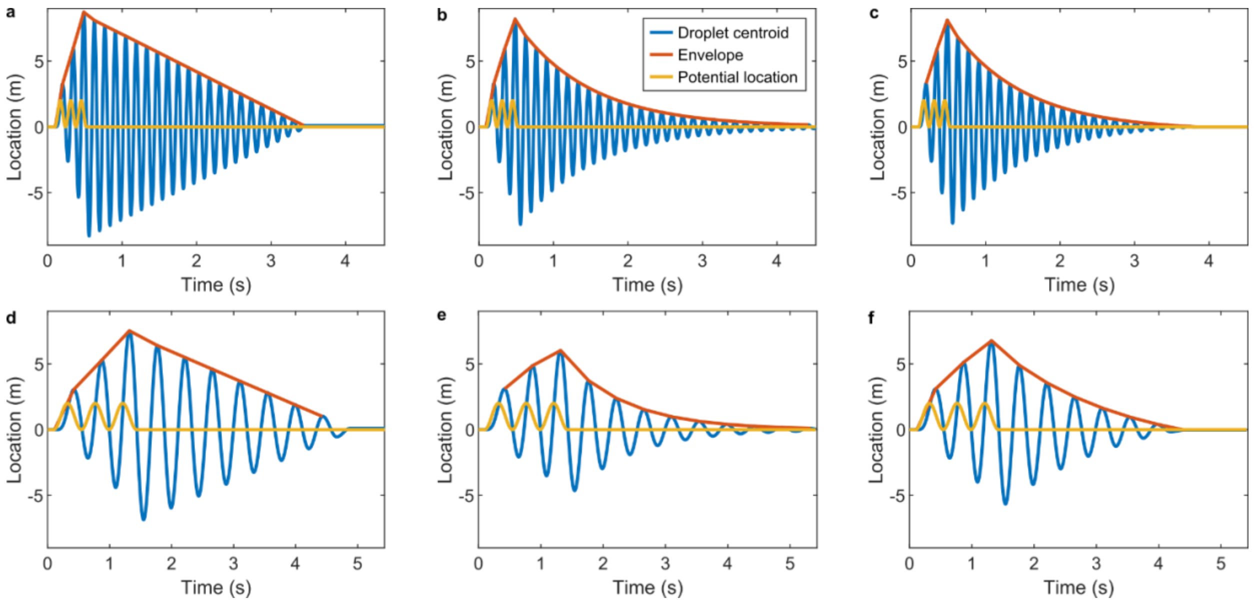

Fig. S13.

Example simulated oscillations with different dissipation forces. The **a** – **c** show example simulated oscillations with droplet mass 1 mg and the **d** – **f** is for 10 mg. The friction force is dominating in **a** and **d**, viscous force is dominating in **b** and **e**, while **c** and **f** have equal contribution from viscous and frictional force. At the start of the oscillations, the potential is moving to cause the droplet to move.

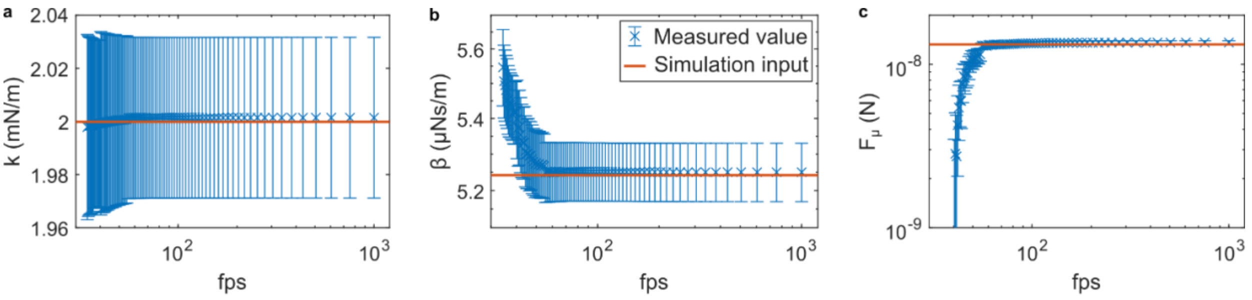

Fig. S14.

Analysis of one simulated data set with different frame rates. The solved spring coefficient (**a**), viscous coefficient (**b**) and friction force (**c**) for one simulated data set with different frame rates. The solved values are accurate within error bars for captured frame rates higher than 60 frames per second (fps) when the frame rate is interpolated to 1000 fps for fitting.

Table S1 | Thickness of black silicon samples.

The average thickness of the etched black silicon (bSi) microstructures as measured with side-view SEM.

Sample	***Structure height, H*** (μm)
bSi A	3.2 ± 0.4
bSi B	2.3 ± 0.1
bSi C	1.23 ± 0.08
bSi D	0.94 ± 0.06
μ_A + bSi A	2.5 ± 0.6
μ_B + bSi A	2.9 ± 0.5

Table S2 | Data used for Fig. 1C.

The scanning MFS micropillar data for **Fig. 1C**. The errors are standard deviations from the time-averages of the force F and contact area diameter $2l$ taken over the time frame used for analysing the sliding friction (see example in **Fig. S2**). The error for the speed comes from the manufacturer of the motor (8% relative error).

***V*, m/s**	**V_{err}, m/s**	**F_{μ}, µN**	**$F_{\mu,err}$, µN**	***2l*, µm**	**$(2l)_{err}$, µm**
0.0005	0.00004	5.3	0.4	993	9
0.001	0.00008	8.2	0.7	1277	12
0.0015	0.00012	7.1	0.7	1240	30
0.002	0.00016	7.4	0.7	1150	20
0.0025	0.0002	7.9	0.5	1158	15
0.003	0.00024	7.4	0.7	1140	10
0.0035	0.00028	7.9	0.6	1321	14
0.004	0.00032	8.1	0.7	1286	12
0.006	0.00048	8.0	0.7	1250	20
0.008	0.00064	7.5	0.8	1263	7
0.015	0.0012	5.1	0.4	933	12
0.015	0.0012	5.4	0.6	910	20
0.01	0.0008	5.2	0.4	865	13
0.015	0.0012	4.7	0.4	838	5
0.0045	0.00036	6.0	0.5	1114	5
0.0055	0.00044	7.4	0.5	1081	10

Table S3 | Data used for Fig. 1C.

The scanning MFS black silicon data for **Fig. 1C**. The errors for the dimensionless force are error propagations using the time-averages and standard deviations of the force F and contact area diameter $2l$ taken over the time frame used for analysing the sliding friction (see example in **Fig. S2**). The surface tension is that of water ($\gamma = 72$ mN/m). The relative error for the speed is 8% as stated by the manufacturer of the motor.

***V*, m/s**	$F_\mu/2l\gamma$	$(F_\mu/2l\gamma)_{err}$
0.015	0.0019	0.0002
0.01	0.00142	0.00011
0.005	0.00101	0.00010
0.001	0.00041	0.00008
0.0005	0.00026	0.00010
0.0001	0.00024	0.00008
0.00005	0.00024	0.00006
0.00005	0.00026	0.00006
0.0001	0.00031	0.00006
0.0005	0.00029	0.00006
0.001	0.00039	0.00009
0.005	0.00105	0.00007
0.01	0.00175	0.00014
0.02	0.0029	0.0004
0.00002	0.00041	0.00004
0.00002	0.00027	0.00003
0.00001	0.00032	0.00003
0.00001	0.00024	0.00004
0.00002	0.00040	0.00004
0.002	0.00055	0.00009
0.002	0.00049	0.00005

Table S4 | Data used for Fig. 2C.

The measured k and calculated k_t spring constants of the oscillating MFS system plotted in **Fig. 2C** with spring constant ($k_p = 2.48 \pm 0.06$ mN/m). The errors for k are the 95% confidence intervals of the ODT fits and the error for k_t is the error propagations of the components (and their standard deviations) in Eq. (1).

***k*, mN/m**	***k*$_t$, mN/m**
1.91 ± 0.09	1.86 ± 0.13
2.00 ± 0.08	1.94 ± 0.14
1.97 ± 0.10	1.83 ± 0.13
1.95 ± 0.08	1.75 ± 0.13
2.0 ± 0.2	1.94 ± 0.14
1.85 ± 0.04	1.61 ± 0.12
2.02 ± 0.07	1.88 ± 0.14
1.93 ± 0.07	1.78 ± 0.13
1.93 ± 0.07	1.73 ± 0.13
1.92 ± 0.03	1.69 ± 0.12
2.25 ± 0.4	2.07 ± 0.15
2.29 ± 0.2	1.78 ± 0.15
2.12 ± 0.04	1.72 ± 0.13
2.14 ± 0.08	2.02 ± 0.15
2.11 ± 0.14	1.57 ± 0.13
2.12 ± 0.05	1.91 ± 0.14
1.91 ± 0.05	1.64 ± 0.12
2.0 ± 0.2	1.64 ± 0.13
2.00 ± 0.12	1.80 ± 0.13
2.1 ± 0.4	1.78 ± 0.14
2.1 ± 0.2	1.85 ± 0.14
2.1 ± 0.2	1.71 ± 0.14
2.1 ± 0.1	1.72 ± 0.14
2.02 ± 0.12	1.73 ± 0.13
1.9 ± 0.2	1.73 ± 0.12
2.0 ± 0.2	1.88 ± 0.14
2.02 ± 0.15	1.63 ± 0.13
1.74 ± 0.05	1.87 ± 0.12
2.05 ± 0.14	1.86 ± 0.14
2.0 ± 0.2	1.96 ± 0.14
2.08 ± 0.13	1.75 ± 0.13
2.1 ± 0.2	1.80 ± 0.13
2.04 ± 0.14	1.81 ± 0.13
2.0 ± 0.2	2.06 ± 0.14
2.11 ± 0.07	2.02 ± 0.14
2.0 ± 0.3	1.81 ± 0.13

Table S5 | Data used for Fig. 2C.

The measured *k* and calculated k_t spring constants of the oscillating MFS system plotted in **Fig. 2C** with spring constant ($k_p = 7.7 \pm 0.2$ mN/m). The errors for *k* are the 95% confidence intervals of the ODT fits and the error for k_t is the error propagations of the components (and their standard deviations) in Eq. (1).

***k*, mN/m**	**k_t, mN/m**
3.20 ± 0.05	3.4 ± 0.2
3.3 ± 0.3	3.6 ± 0.2
3.29 ± 0.08	3.6 ± 0.2
3.51 ± 0.14	3.6 ± 0.2
3.51 ± 0.06	3.8 ± 0.2

Table S6 | Data used for Fig. 2C.

The measured k and calculated k_t spring constants of the oscillating MFS system plotted in **Fig. 2C** with spring constant ($k_p = 10.8 \pm 0.4$ mN/m). The errors for k are the 95% confidence intervals of the ODT fits and the error for k_t is the error propagations of the components (and their standard deviations) in Eq. (1).

***k*, mN/m**	***k*$_t$, mN/m**
5.8 ± 1.4	5.2 ± 0.4
6.6 ± 1.9	5.8 ± 0.4
8.0 ± 2.2	7.0 ± 0.6
8.0 ± 0.8	7.6 ± 0.6
5.9 ± 1.9	6.3 ± 0.4
6.7 ± 0.6	7.0 ± 0.5
6.2 ± 0.2	6.5 ± 0.5
6.0 ± 0.9	5.9 ± 0.4
5.7 ± 1.0	5.8 ± 0.4
4.6 ± 0.3	4.2 ± 0.3
5.0 ± 0.3	5.2 ± 0.3
5.1 ± 0.4	4.7 ± 0.3
5.6 ± 0.2	5.7 ± 0.4
5.6 ± 0.5	5.8 ± 0.4
5.0 ± 0.5	4.8 ± 0.3
4.4 ± 0.7	5.3 ± 0.3
4.7 ± 1.0	4.9 ± 0.3
4.1 ± 1.1	5.1 ± 0.3
5.0 ± 0.7	4.6 ± 0.3
4.8 ± 0.9	4.9 ± 0.3
4.8 ± 0.3	4.3 ± 0.3
4.9 ± 0.7	5.2 ± 0.3
4.8 ± 1.1	4.7 ± 0.3
4.9 ± 0.5	5.2 ± 0.3
5.5 ± 0.3	5.0 ± 0.4

Table S7 | Data used for Fig. 3B.

The gas film height as a function of radial distance as measured with RICM for **Fig. 3B**.

***r*, µm**	***h*, µm**
0	3.6
95.9	3.3
136.2	3.0
168.2	2.7
193.2	2.4
215.5	2.1
234.9	1.8
253.0	1.5
271.1	1.1
287.8	0.8
305.8	0.5
337.8	0.2
364.2	0.5
371.2	0.8
378.1	1.1
382.3	1.5
386.5	1.8
390.7	2.1
394.8	2.4

Table S8 | Data used for Fig. 3C.

The calculated β_{cushion} and measured β damping coefficient of the oscillating MFS measurements on the carbonated drops plotted in **Fig. 3C**. The errors for β are the 95% confidence intervals of the ODT fits and the errors for β_{cushion} are the error propagations of the components (and their standard deviations) in Eq. (2).

$\boldsymbol{\beta_{cushion}}$, µNs/m	$\boldsymbol{\beta_{cushion,err}}$, µNs/m	$\boldsymbol{\beta}$, µNs/m	$\boldsymbol{\beta_{err}}$, µNs/m
5.7	0.9	7.7	1.5
5	2	10.0	0.9
4.4	0.9	8.7	1.1
6.8	1.5	10.1	0.6
3.3	1.1	6.0	0.4
5	2	7.2	0.7
2.6	0.7	4.8	0.5
3.8	0.7	6.5	0.5
1.7	0.9	3.6	0.2
1.4	0.8	3.0	0.3
3.1	0.7	4.1	0.4
7	2	13.3	0.9
7	3	12.8	1.1
6	3	12.5	0.9
6	3	10.7	1.0
2.9	0.6	5.8	0.6
2.5	1.2	3.67	0.05
3.0	1.3	5.06	0.12
4	2	8.7	0.3
2.9	1.3	7.2	0.4
4	2	9.0	0.3

Table S9 | Data used for Fig. 4F.

The contact area radius l and damping coefficient β data from the oscillating MFS measurements with water drops on **bSi A** plotted in **Fig. 4F**. The errors for β are the 95% confidence intervals of the ODT fits and the errors for l are the standard deviation of the time-averaged distance measurement.

l, µm	l_{err}, µm	β, µNs/m	β_{err}, µNs/m
520	20	23.3	0.8
350	40	11.7	0.4
410	40	15.4	0.9
460	30	20.9	1.6
360	20	14.2	0.5
250	10	5.1	0.6
300	50	8.9	1.0
230	30	5.5	0.7

Table S10 | Data used for Fig. 4F.

The contact area radius l and damping coefficient β data from the oscillating MFS measurements with water drops on **bSi B** plotted in **Fig. 4F**. The errors for β are the 95% confidence intervals of the ODT fits and the errors for l are the standard deviation of the time-averaged distance measurement.

l, µm	l_{err}, µm	β, µNs/m	β_{err}, µNs/m
196	3	4.8	1.3
200	30	5.0	0.5
189	3	4.0	0.5
160	10	3.4	0.4
274	4	7.7	0.6
360	20	15	2
423	14	18.4	0.5
470	70	24.1	1.3
420	20	24.4	1.3
466	7	24.4	1.3
539	3	30.3	0.6

Table S11 | Data used for Fig. 4F.

The contact area radius l and damping coefficient β data from the oscillating MFS measurements with water drops on **bSi C** plotted in **Fig. 4F**. The errors for β are the 95% confidence intervals of the ODT fits and the errors for l are the standard deviation of the time-averaged distance measurement.

l, µm	l_{err}, µm	β, µNs/m	β_{err}, µNs/m
199	6	11.3	1.4
180	20	6.3	1.1
132	7	5.1	0.6
150	20	5.7	1.0
258	3	15.1	2.7
340	20	27.5	5.9
420	20	47	12
400	20	53	13

Table S12 | Data used for Fig. 4F.

The contact area radius l and damping coefficient β data from the oscillating MFS measurements with water drops on **bSi D** plotted in **Fig. 4F**. The errors for β are the 95% confidence intervals of the ODT fits and the errors for l are the standard deviation of the time-averaged distance measurement.

l, µm	l_{err}, µm	β, µNs/m	β_{err}, µNs/m
257	7	23	4
200	20	17	2
180	7	14	3
219	14	14	4
327	7	56	6
361	10	53	11
490	30	84	7
178	7	15	1

Table S13 | Data used for Fig. S8.

The contact area radius l and damping coefficient β data from the oscillating MFS measurements with water drops on **μ_A+bSi A** plotted in **Fig. S8**. The errors for β are the 95% confidence intervals of the ODT fits and the errors for l are the standard deviation of the time-averaged distance measurement.

l, µm	l_{err}, µm	β, µNs/m	β_{err}, µNs/m
340	10	0.1	2
420	50	0.4	2
480	30	1	3
257	14	0.4	1.4

Table S14 | Data used for Fig. S8.

The contact area radius l and damping coefficient β data from the oscillating MFS measurements with water drops on **μB+bSi A** plotted in **Fig. S8**. The errors for β are the 95% confidence intervals of the ODT fits and the errors for l are the standard deviation of the time-averaged distance measurement.

l, µm	l_{err}, µm	β, µNs/m	β_{err}, µNs/m
314	2	1.9	1.1
225	1	1.3	1.2
399	7	4.1	1.2
470	50	6	2
380	20	3	2
403	10	5.1	1.3
320	20	3.5	1.1
270	20	2.9	0.9

Table S15 | Data used for Fig. 5D.

The scanning MFS bSi data from sample **μ_A+bSi A** in **Fig. 5D**. The errors for the dimensionless force are error propagations using the time-averages and standard deviations of the force *F* and contact area diameter *2l* taken over the time frame used for analysing the sliding friction (see example in **Fig. S2**). The surface tension is that of water ($\gamma = 72$ mN/m). The relative error for the speed is 8% as stated by the manufacturer of the motor.

V, m/s	$F_\mu/2l\gamma$	$(F_\mu/2l\gamma)_{err}$
0.0001	0.0005	0.0002
0.0005	0.0008	0.0004
0.00005	0.0007	0.0002
0.001	0.0006	0.0004
0.01	0.0006	0.0003
0.00001	0.00048	0.00011

Table S16 | Data used for Fig. 5D.

The scanning MFS bSi data from sample **$μ_B$+bSi A** in **Fig. 5D**. The errors for the dimensionless force are error propagations using the time-averages and standard deviations of the force (*F*) and contact area diameter *2l* taken over the time frame used for analysing the sliding friction (see example in **Fig. S2**). The surface tension is that of water ($\gamma = 72$ mN/m). The relative error for the speed is 8% as stated by the manufacturer of the motor.

***V*, m/s**	$F_\mu/2l\gamma$	$(F_\mu/2l\gamma)_{err}$
s0.0001	0.00045	0.00010
0.01	0.00063	0.00012
0.015	0.00087	0.00012
0.02	0.0013	0.0002
0.001	0.00051	0.00009
0.0005	0.00038	0.00014
0.00005	0.00044	0.00009
0.005	0.0005	0.0002
0.01	0.00053	0.00010
0.005	0.00057	0.00015
0.001	0.0003	0.0002
0.0005	0.00028	0.00013
0.0001	0.00058	0.00013
0.00005	0.00036	0.00011
0.00001	0.00051	0.00010
0.015	0.0012	0.0002
0.02	0.0012	0.0002
0.01	0.00071	0.00014

Movie S1 | Side-view movie of an oscillating MFS experiment.

Side-view movie (SuppMov1.mp4) of an oscillating micropipette force sensor experiment with a carbonated water drop ($R = 1.12 \pm 0.03$ mm, $l = 350 \pm 1$ μm; same as in **Fig. 2a** in the main text) on Glaco. The movie is slowed down 4 times.

Movie S2 | Bottom-view movie of an oscillating MFS experiment.

Bottom-view movie (SuppMov2.mp4) using reflection interference contrast microscopy of the contact region between the carbonated water drop and the Glaco surface (same as in **Fig. 2a** in the main text). The time-range of the movie corresponds to that analysed with oscillating droplet tribology in **Fig. 2a** in the main text.

Movie S3 | Bottom-view movie (long version) of an oscillating MFS experiment.

Bottom-view movie (SuppMov3.mp4) using reflection interference contrast microscopy of the contact region between the carbonated water drop and the Glaco surface from the entire experiment in **Fig. 2a** in the main text, showing the collapse of the CO_2 film at the end.
